# Multiple genomic regions influence root morphology and seedling growth in cultivated sunflower (*Helianthus annuus* L.) under well-watered and water-limited conditions

**DOI:** 10.1371/journal.pone.0204279

**Published:** 2018-09-20

**Authors:** Rishi R. Masalia, Andries A. Temme, Nicole de leon Torralba, John M. Burke

**Affiliations:** Department of Plant Biology, University of Georgia, Athens, Georgia, United States of America; Università Politecnica delle Marche, ITALY

## Abstract

With climate change and an ever-increasing human population threatening food security, developing a better understanding of the genetic basis of crop performance under stressful conditions has become increasingly important. Here, we used genome-wide association studies to genetically dissect variation in seedling growth traits in cultivated sunflower (*Helianthus annuus* L.) under well-watered and water-limited (i.e., osmotic stress) conditions, with a particular focus on root morphology. Water limitation reduced seedling size and produced a shift toward deeper rooting. These effects varied across genotypes, and we identified 13 genomic regions that were associated with traits of interest across the two environments. These regions varied in size from a single marker to 186.2 Mbp and harbored numerous genes, some of which are known to be involved in the plant growth/development as well as the response to osmotic stress. In many cases, these associations corresponded to growth traits where the common allele outperformed the rare variant, suggesting that selection for increased vigor during the evolution of cultivated sunflower might be responsible for the relatively high frequency of these alleles. We also found evidence of pleiotropy across multiple traits, as well as numerous environmentally independent genetic effects. Overall, our results indicate the existence of genetic variation in root morphology and allocation and further suggest that the majority of alleles associated with these traits have consistent effects across environments.

## Introduction

Plants are faced with a variety of abiotic challenges throughout their lives. Water limitation is perhaps the most important of these stressors, with drought having an enormous impact on crop yields and agricultural sustainability [1–4]. Unfortunately, this challenge is expected to become more pervasive and severe as climate change produces increasingly unpredictable precipitation patterns resulting in longer and more frequent periods of drought [5–7]. In an agricultural setting, water limitation inhibits seedling establishment and reduces plant growth rates, thereby reducing yields [3,8,9]. When faced with water limitation, plants respond in a variety of ways, including reduced stomatal conductance and photosynthesis [10,11], increased resource allocation towards roots for improved resource acquisition [12–15], the accumulation of organic solutes and/or inorganic ions for osmotic adjustment [3,16–20], and detoxification of reactive oxygen species (ROS) to combat oxidative damage under stress [21,22].

Responses to water limitation are genetically complex due to the involvement of numerous biochemical, molecular, and physiological mechanisms affecting plant growth and development [3,4,23]. Recent years have seen increased interest in determining the functional basis of variation in plant performance under stress [24,25]. This has included both detailed physiological studies (e.g., [26–28]) as well as genomic analyses aimed at identifying genetic variants underlying observed trait variation (e.g., [29–31]). Such efforts have been motivated, at least in part, by a desire to better understand the ways in which plants handle water limitation as a step toward developing increasingly resilient crop plants [3,4,22].

Of particular interest to many researchers has been the relationship between root-related traits and performance under water limitation. It has been observed that plants often increase their root biomass allocation and alter their root morphology under water limitation, presumably to improve water uptake from the soil (e.g., [10,15,32–34]; but see [13] for a discussion of allometric scaling biases). Recent years have seen significant progress in understanding the genetic basis of this trait variation. Indeed, numerous studies have reported the identification of quantitative trait loci (QTL) underlying traits associated with increased root foraging, including root length [35–38], root biomass [38,39], lateral root length [40], and root angle [29,33,41–45].

In most cases, root traits have been found to be under complex genetic control, with multiple QTL of small to moderate effect [15,46]. Moreover, these traits often exhibit correlations with aboveground traits (e.g., [39,47,48]) as well as across environments [46,49–51]. These correlations may be due to either genetic linkage or pleiotropy [48,52], and they can be synergistic or antagonistic across traits and/or environments. That is, variants conferring improved performance in terms of a particular trait or environment may come at the cost of performance in another trait or environment. These sorts of antagonistic correlations have often been observed for traits/QTL related to stress resistance (e.g., [3,24,53]), though others have argued that high performance under ideal conditions is predictive of relatively high performance under stressful conditions (e.g., [53–55]). Here we describe the use of genome-wide association (GWA) studies to investigate the genetic architecture of variation in root morphology, and its relationship to seedling growth in cultivated sunflower (*Helianthus annuus* L.) under well-watered vs. water-limited conditions.

Cultivated sunflower, which is one of the world’s most important oilseed crops, is a deep-rooted plant that has the potential to tap soil moisture reserves that are inaccessible to many other crops [56,57]. It is, however, often grown as a rainfed crop, and water limitation at the seedling stage can severely reduce stand establishment and negatively impact yields [8,58]. In this study, water limitation was induced as an osmotic challenge, which provides a means for limiting water availability to plants in a uniform and repeatable way. We measured several root and growth traits and used GWA studies to identify genomic regions underlying variation in these traits in two distinct environments (i.e., well-watered vs. water-limited). Our goals were to: (1) determine the phenotypic response of sunflower seedlings to water limitation, imposed as an osmotic stress; (2) characterize the genetic basis of trait variation under well-watered and water-limited (i.e., control and stressed) conditions; (3) investigate the extent to which alleles underlying the observed trait variation had effects across traits within the same treatment and/or across treatments; and (4) identify candidate genes underlying significant associations.

## Materials and methods

### Association mapping population

We used the sunflower association mapping (SAM) population developed by [59] and described more fully by [60]. This population, which is derived from the *H*. *annuus* germplasm collections maintained by the USDA’s North Central Regional Plant Introduction Station (NCRPIS) and the French National Institute for Agricultural Research (INRA), is composed of 288 lines that capture ca. 90% of the allelic diversity present within the cultivated sunflower gene pool (S1 Table). It includes accessions from both major heterotic groups (i.e., HA and RHA), as well as both market types (i.e., oilseed and confectionery).

### Experimental design

In the fall of 2015, we germinated individual sunflower seedlings from the SAM population and planted them in three replicates across two treatments (i.e., control and stressed; n = 3 replicates × 2 treatments × 288 lines = 1,728 individual seedlings) in a growth room in the Miller Plant Sciences Building at the University of Georgia (Athens, GA). Due to space constraints, the three biological replicates were grown sequentially, with all entries being planted in a completely randomized fashion within treatments. For each replicate, eight seeds per line were scarified to promote germination, placed on damp filter paper in a Petri dish, and kept in the dark. After 24 hours, seed coats were removed and the seeds were returned to the dark. After 24 additional hours, the Petri dishes were brought out into the light for 72 hours and, five days post-scarification, two viable seedlings (one per treatment) were selected for transplant into individual 50 mL Falcon tubes containing beach sand as a growth substrate. Prior to transplanting, the Falcon tubes were pre-drilled with two ⅛-inch holes 2 cm from the base to allow drainage.

To accommodate the 576 seedlings per replicate, the experiment was split into four trays per treatment, with each tray containing 72 randomly assigned lines. These trays were arranged in an alternating fashion by treatment in the growth room to minimize variation across treatments. After planting, all tubes were top-watered daily with a solution of deionized (DI) water and one g/L of Jack’s All Purpose 20-20-20 aqueous mix (J.R. Peters, Inc., Allentown, PA) to facilitate establishment. Following establishment, seedlings were separated into the two treatments: the well-watered control (conditions maintained as above) and the water-limited (stressed) scenario. Water limitation was implemented as an osmotic stress using polyethylene glycol (PEG-6000, 8.25% by volume; hereafter referred to as PEG), which is a high molecular weight polymer that induces an osmotic challenge [61,62]. The use of high molecular weight polymers such as PEG is a generally accepted approach for inducing water limitation (e.g., [63–67]) as they do not enter cellular pores [62,68] and are thus less prone to the toxic effects elicited by low molecular weight osmotica (but see [69] for evidence that such compounds can influence root hair elongation). They thus induce cytorrhysis, similar to what occurs under drought conditions, rather than plasmolysis as is seen with low molecular weight osmotica [62,70]. Seedlings in the stressed treatment were transitioned from control conditions during establishment to stressed conditions by flushing the growth substrate with a solution containing 8.25% PEG with one g/L of Jack’s All Purpose 20-20-20 aqueous solution in DI water. This solution produced an initial osmotic challenge of -0.25 MPa as measured using a vapor pressure osmometer (Vapro 5520; Wescor, Inc., Logan, UT). This treatment was then maintained by flushing the growth substrate with fresh solution each day. Note, however, that we did not explicitly monitor the PEG concentration in the growth substrate for the duration of the experiment.

Throughout the experiment, the temperature for both treatments was kept at 20°C with a 16h:8h light:dark cycle. Seedlings were harvested and subjected to detailed phenotypic characterization seven days after initiation of the differential watering regime. This procedure was then repeated two more times for the second and third replicates.

### Phenotypic analyses

A total of 12 morphological traits were measured for each individual. These included stem, leaf, and root traits, as well as metrics of overall growth. At harvest, seedlings were gently uprooted and their roots were rinsed to remove soil substrate. Stem height and diameter (just above the soil line) were measured using Fowler 6"/150mm Ultra-Cal IV Electronic Calipers (Fowler Tools and Instruments, Newton, MA). The seedlings were then divided into their component parts by removing all leaves and cotyledons as well as intact root tissue. Stem, leaf, and cotyledon tissue was dried at 60°C for 72 hours in a forced air drying oven and then weighed to determine biomass and biomass fractions.

**Fig 1:**
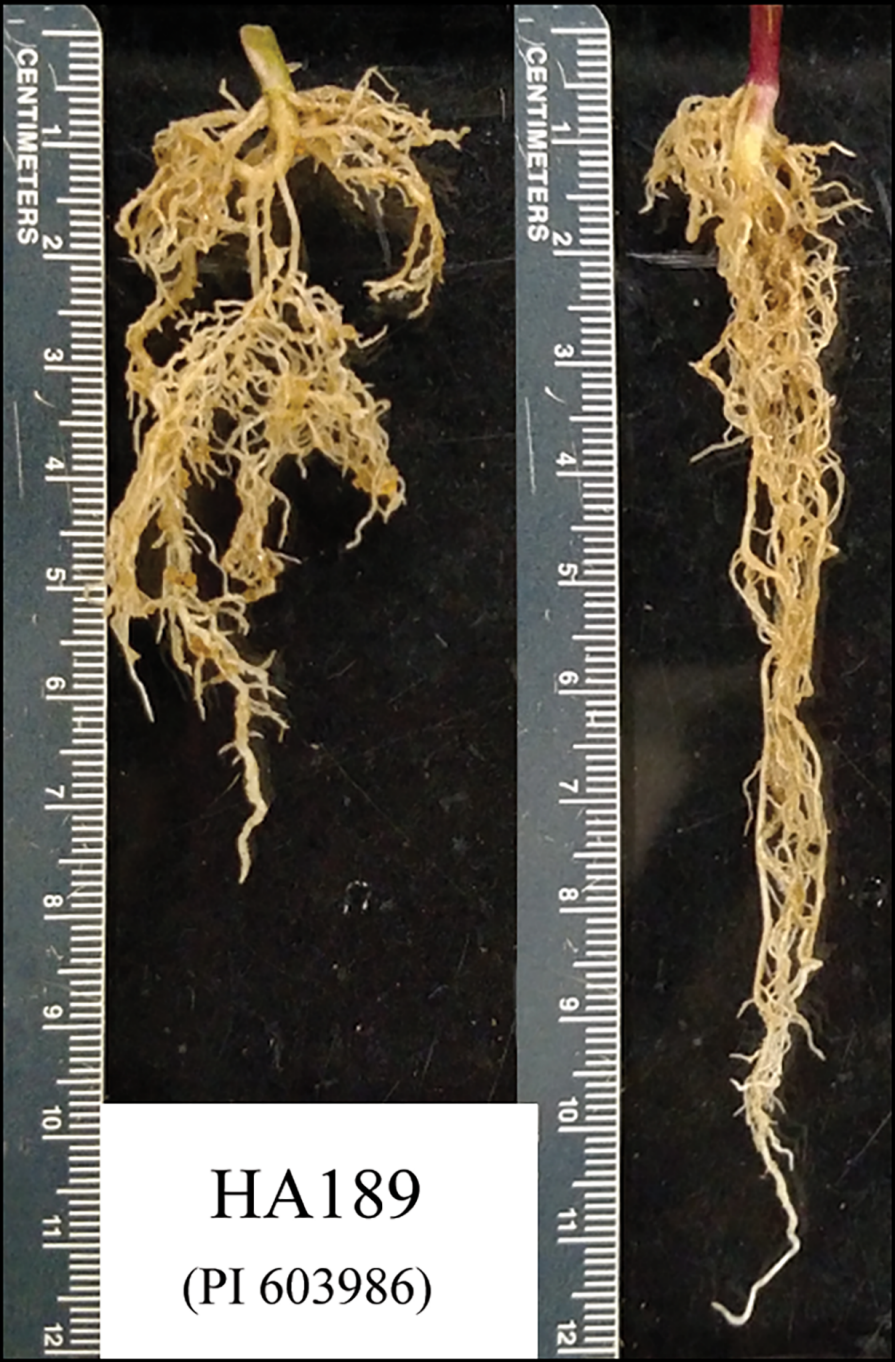
Example of the growth response of a single genotype from the SAM population grown under well-watered (left) and water-limited (right) conditions. Note that the pigmentation at the base of the stem in the seedling on the right is an apparent stress response that was anecdotally observed in a subset of genotypes during the course of this work.

Intact root systems were stained with toluidine blue for four minutes to facilitate image analysis. Stained roots were then measured for taproot length using a ruler, and severed 2 cm below the emergence of the first basal root, to establish sections for upper and lower root allocation, (see Fig 1 for root images of an example genotype across both treatments). The 2 cm mark was chosen as the point of separation as most lateral root development in sunflower seedlings occurs at or above this point [71]. Both the upper and lower root sections were scanned with a CanoScan 8800F flatbed desktop scanner (Canon USA, Inc., Melville, NY) at 600 dpi and analyzed using the root imaging program WinRhizo.v.2 (Regent Instruments, Quebec, Canada; [72]. A manual threshold setting of 80 was used to separate root pixels from the background, while the ratio of pixels to physical distance was calibrated using a scanned ruler. Total root length (TRL) was then determined separately for each root allocation section. Roots were dried and weighed as above to determine root biomass for each root allocation section. Total root length and root biomass values for each allocation section were then summed to determine the values for the entire root system, while TRL allocation and root biomass allocation were determined by dividing the values from the upper portion by the total. Finally, specific root length (SRL) was calculated for the entire root system by dividing TRL by root biomass.

Overall seedling growth was investigated using biomass production. Total biomass was calculated as the sum of the dried leaf, stem, and root tissue. Organ biomass fractions were also determined from these data. It should be noted, however, that total and aboveground biomass traits as well as leaf mass fraction (LMF) and stem mass fraction (SMF) were increased under stressed conditions, while root mass fraction (RMF) was similar under both treatments; all of these traits also had significant line-by-treatment interactions (*P* < 0.001). While this treatment effect could be a real biological phenomenon, we observed an accumulation of PEG residue on the aboveground tissues, likely due to droplets of the PEG solution having dried onto these tissues following watering. Notably, at a PEG concentration of 82.5 g/L, the evaporation of one mL of solution would leave behind 82.5 mg of residue, so even a few drops of dried PEG solution could produce a measurable increase in mass. The belowground tissues were not subject to such accumulation, as the soil was kept constantly wet throughout the experiment and the root tissues were carefully washed prior to imaging. Thus, to protect against a potential methodological artifact in terms of biomass estimation, we did not analyze aboveground or total biomass values any further. Instead, we used stem height and diameter as proxies for overall plant performance; stem diameter in particular is known to be a good predictor of biomass accumulation in sunflower [73].

### Statistical analysis of phenotypic traits

All phenotypic data analyses were performed in R v3.2.4 [74]. To test for line-by-treatment interactions, a linear mixed model was run using the “lme4” package [75], with line and treatment as fixed effects and a treatment-by-tray interaction nested within replicate and treated as a random effect. The only trait for which the model residuals showing a skewed distribution based on a Shapiro-Wilk test of normality was SRL [76], so those trait values were log-transformed prior to analysis. For all traits, least-squares means [77] for each treatment were calculated based on the best-fitting model for all lines, and these values were used for association mapping. Least-squares mean values were also used to calculate a trait-based principal component analysis (PCA) grouped by treatment [78], as well as a series of trait-trait correlations both within and across treatments using the corrplot package [79]. To account for multiple comparisons, significance values for the trait-trait correlations were adjusted using a Bonferroni correction (α = 0.05 / the total number of trait-trait correlations tested).

### Genotypic characterization and genome-wide association studies

Genotypic information for the SAM lines was extracted from available whole-genome shotgun sequencing data (NCBI Sequence Read Archive, Bioproject PRJNA353001). Briefly, following sequence alignment against the HanXRQ sunflower genome assembly [80], single nucleotide polymorphisms (SNPs) were called using Genome Analysis Toolkit (GATK v3.8–0; Broad Institute, Boston, MA). Before finalizing our SNP calls, however, we removed 24 lines that exhibited higher than expected levels of residual heterozygosity based on preliminary analyses. The analysis of the remaining 264 lines resulted in the identification of an initial set of 1.81M putative polymorphisms spanning the 17 chromosomes and estimated 3.6 Gbp length of the sunflower genome. We further reduced the dataset to account for missing data due to seedling mortality, resulting in a final population of 213 lines. The initial SNP set was then filtered to remove markers with ≥ 30% missing data (after converting remaining heterozygous loci to missing data) and ≤ 5% minor allele frequency (MAF).

Association mapping was conducted for all phenotypic traits in both treatments using a collection of custom R scripts (the Enchilada Suite; https://github.com/masalia). We used the Efficient Mixed-Model Association eXpedited (EMMAX) algorithm [81], which accounts for both population structure (*Q)* and familial relatedness (i.e., kinship; *k*), to test for significant associations. Model effects and likelihood values for individual SNPs were extracted from the EMMAX output, and Manhattan plots were generated using the R package qqman [82]. These steps were automated using a custom script (Enchilada Suite: Burrito). We chose EMMAX over the alternatives due to its computational efficiency, which allows for the rapid processing of numerous trait and treatment combinations. Population structure (*Q*) for all 213 lines was estimated based on the entire filtered SNP set using the bioconductor package SNPRelate [83], and an identity-by-state (IBS) kinship (*k*) matrix was estimated via EMMAX for all 213 lines using all filtered SNPs. Both *Q* and *k* were then incorporated into our GWA studies to protect against spurious associations.

Given the non-independence of linked markers within the dataset, a traditional Bonferroni correction to protect against false-positives due to multiple comparisons would result in overly conservative significance thresholds. We therefore used the PLINK v1.9 indep-pairwise linkage disequilibrium (LD) variant pruning function [84] to estimate the effective number of independent tests being run. This was done using a window size of 100 kbp with a SNP step size of 10 and an *r*^2^ threshold of 0.8, and the resulting value was used to adjust the significance threshold (α = 0.05 / estimated number of independent tests). It should, however, be kept in mind that the lack of a detectable effect for a given trait/treatment could be a false negative due to the highly conservative correction for multiple comparisons employed herein and/or the relatively limited sample size. To further explore this possibility, we identified the most significant SNP (i.e., the one with the lowest *P*-value) within each associated region and queried its *P*-value for other traits within the same treatment as well as the same trait in the alternate treatment. These *P*-values were then converted into a percentile rank amongst all SNPs for the alternate trait/treatment of interest to provide an indication of the likelihood that the region of interest had additional phenotypic (i.e., pleiotropic) effects. While this approach does not protect against the outright failure to detect a real genetic effect, it helps protect against incorrectly concluding that a significantly associated region has no effect on other traits, or in the alternate treatment.

Finally, the relative effect size of an associated region on a particular trait in a given treatment was estimated as the follows:

Relativeeffectsize=|(2xβ/rangeofobservedtraitvalues)x100%|

where β is effect size estimate of the major allele (as determined by EMMAX) and the range of observed trait values was calculated as the maximum minus the minimum value of the LS mean values of the trait of interest (across all 213 genotypes) per a given treatment. As such, the effect size represents the percentage of the observed range of variation in a trait that can be explained by a particular association in the treatment of interest.

### Linkage disequilibrium and the extent of associated regions

Rather than treating all significant SNPs as independent associations, we delineated the boundaries of significantly associated genomic regions based on observed patterns of LD using LDSelect v1.0 [85]. This involved collapsing significant SNPs along a chromosome for a given trait/treatment combination into bins based on a default threshold of *r*^2^ ≥ 0.80. This threshold has previously been found to be sufficient for defining independent blocks in association studies (e.g., [85–88]). We further examined all suggestive SNPs (i.e., SNPs with *P*-values in the top 5% of all values for a given trait/treatment combination) along a chromosome to determine reasonable boundaries for each associated region. This allowed us to extend significant regions based on observed patterns of LD, rather than restricting our focus to intervals demarcated by SNPs that exceeded the highly conservative significance thresholds employed herein. Significantly associated regions were thus defined as spanning contiguous blocks of significant SNPs plus any suggestive SNPs that collapsed into these blocks based on the LD threshold (i.e., *r*^2^ ≥ 0.80). To visualize these blocks, we estimated *r*^2^ values between all possible pairs of significant and suggestive SNPs for each associated region using PLINK v1.9 with default settings and displayed the results using ggplot2 [78] in R (Enchilada Suite: SALSA).

### Candidate gene identification

We used a custom script (Enchilada Suite: GUAC) to compile a list of genes underlying each associated region using the annotation of the HanXRQ sunflower genome assembly v1.2 [80]; https://www.heliagene.org/HanXRQ-SUNRISE/). To protect against the exclusion of potentially relevant genes near the boundaries of an associated region, we included an additional gene flanking both the beginning and end of the region. This feature was implemented as a “buffer” flag (*b*), with a default setting of one. This results in a list of all genes within the associated region plus (2 × *b*) additional genes. For single marker associations, the list thus included (2 × *b*) genes if the marker fell between genes, or 1 + (2 × *b*) genes if it fell within a gene.

## Results

### Phenotypic response of sunflower seedlings to osmotic stress

**Fig 2:**
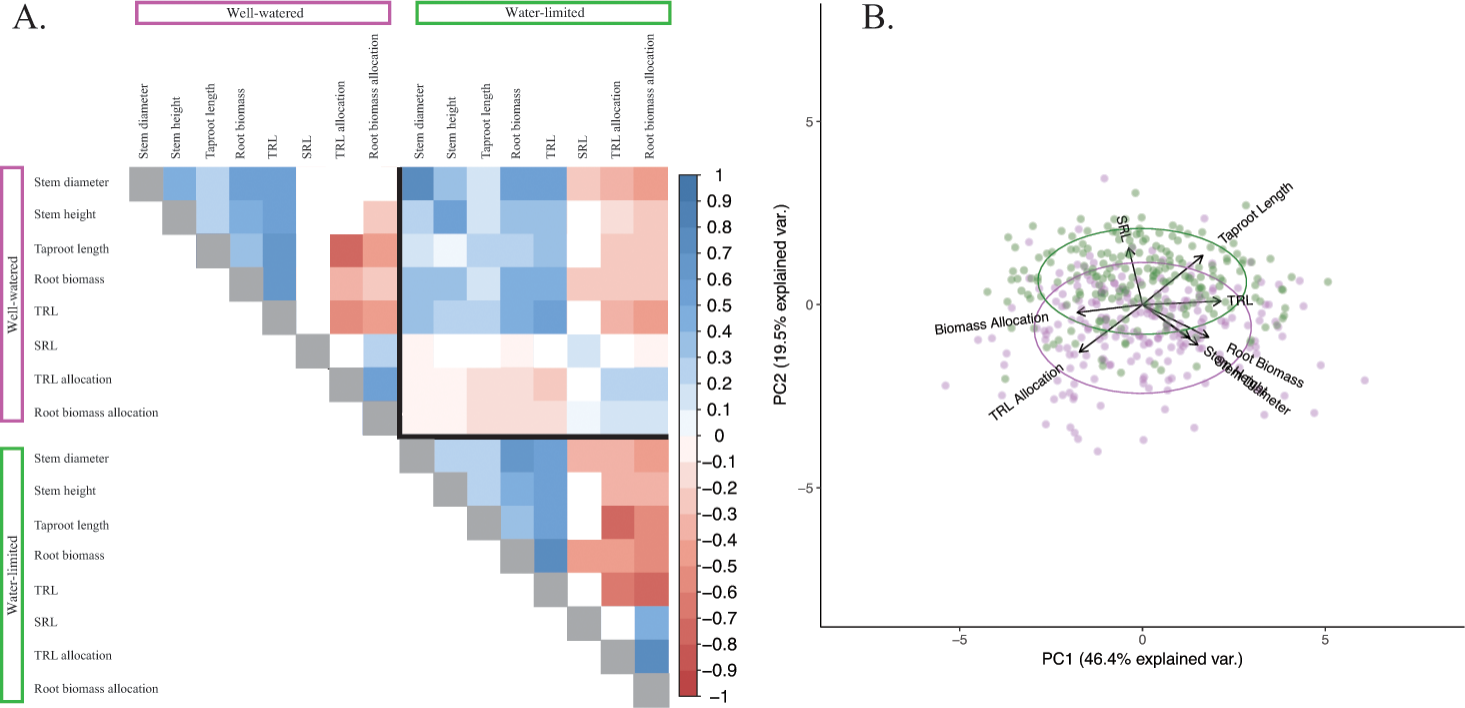
Phenotypic response of sunflower seedlings to osmotic stress. (A) Trait-trait correlations within and across treatments. Traits grouped by a purple line are from the well-watered treatment, while traits grouped by the green line are from the water-limited treatment. Colored boxes correspond to correlations that are significant after correcting for multiple corrections, with color and gradient indicating direction and strength of correlation. (B) First two principal components of principal component analysis with all traits (n = 8). Individual genotypes are shown as circles and are represented twice in the figure, once per treatment. Color denotes treatment, with purple being well-watered and green being water-limited.

All measured traits had significant line-by-treatment interactions (all *P* < 0.0001; Table 1). As an indication of overall plant performance, both stem height and diameter were reduced under osmotic stress, resulting in smaller seedlings. Under control conditions, these aboveground traits were significantly (*P* < 0.001) and positively correlated with each other, as well as with taproot length, root biomass, and TRL. In contrast, taproot length, root biomass, and TRL were all negatively correlated with root biomass allocation and TRL allocation. Within the stressed treatment, many of these relationships held, though there were more significant correlations, including SRL being negatively correlated with stem diameter and root biomass. Across treatments, all traits were significantly and positively correlated with themselves under the alternate conditions, and similar trait-trait correlations to those seen within treatments also held across treatments (Fig 2A; S2 Table).

**Table 1:** The unadjusted phenotypic means and standard error of all traits (n = 8). Mixed linear model results are shown as F-values. Root biomass allocation and total root length allocation are the distribution of mass and length between the top 2 cm and bottom of root tissue.

Traits	Trait mean ± std. error		Mixed linear model results		
	Well-watered	Water-limited	Line	Treatment	Line:Treatment
Stem Diameter (mm)	2.41 ± 0.02	2.26 ± 0.02	5.6848***	59.3425***	0.9307**
Stem Height (mm)	44.3 ± 0.87	34.4 ± 0.69	4.1104***	32.6745***	1.0814***
Taproot Length (mm)	102.6 ± 1.4	113.9 ± 1.2	2.0341***	5.3574*	1.0868***
Root Biomass (mg)	8.2 ± 0.20	7.9 ± 0.20	2.6545***	2.4029	0.8774**
Total Root Length (mm)	1475.53 ± 35.45	1517.39 ± 35.67	1.5760***	0.0297	1.0829***
Specific Root Length (mm/g)	12.87 ± 0.04	13.17 ± 0.02	3.7132***	0.0915	0.9991***
Root Biomass Allocation	0.69 ± 0.01	0.68 ± 0.01	1.7568***	14.8772***	1.0351***
Total Root Length Allocation	0.4 ± 0.01	0.33 ± 0.01	1.4104***	5.1598*	1.0153***

*P < 0.05

**P < 0.001

***P < 0.0001 for treatment effect

The PCA shows the overall phenotypic separation of seedlings by treatment (Fig 2B). Here, root biomass and root biomass allocation, as well as TRL and TRL allocation loaded most strongly along PC1, which explained 46.4% of the observed variation, and tended to reflect variation amongst lines within treatments. In contrast, TRL allocation, stem diameter, SRL, and taproot length loaded most strongly on PC2, which explained 19.5% of the observed variation, and tended to reflect differentiation between treatments. Root biomass allocation (i.e., top root biomass / total root biomass) and stem diameter increased under control conditions, while SRL and taproot length increased under stressed conditions.

### Genotypic characterization and genome-wide association studies

SNPRelate identified 27 principal components corresponding to population structure (*Q*). The first four, which accounted for 7.2%, 6.2%, 4.1%, and 3.1% of the total genotypic variation, respectively, are shown in S1 Fig. The identity-by-state kinship matrix for all 213 genotypes is presented as a heatmap in S2 Fig.

**Fig 3:**
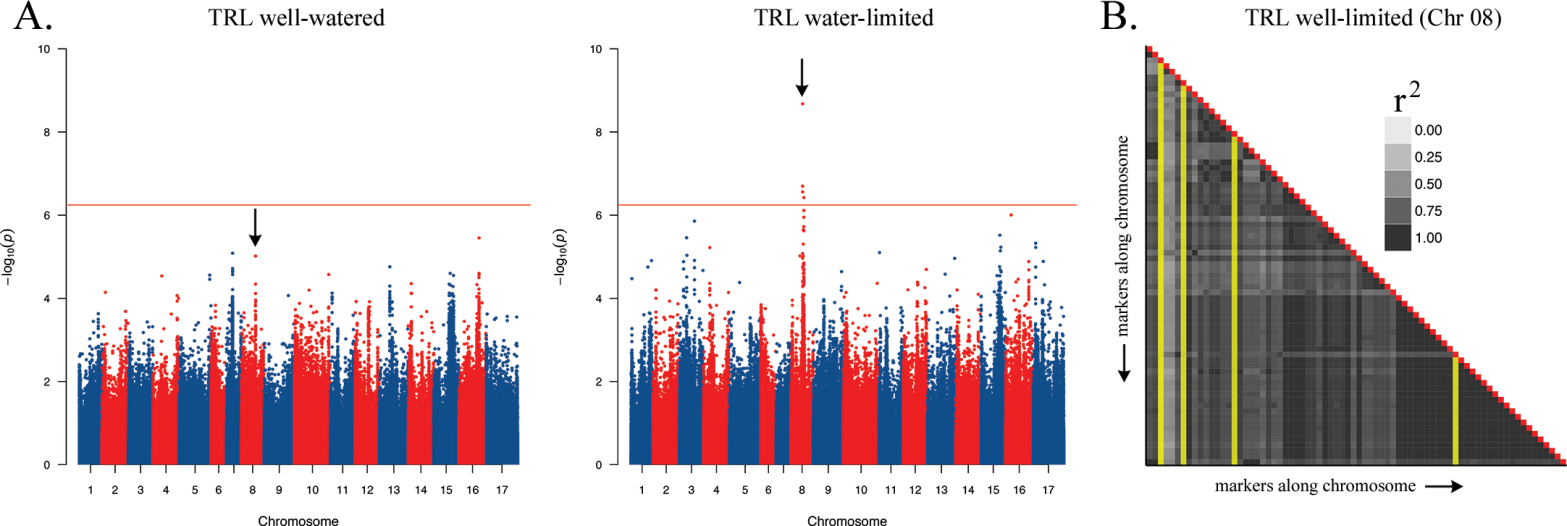
(A) Genome-wide association results for TRL under well-watered (left) and water-limited (right) conditions. Colors alternate by chromosome, dots correspond to SNPs, and the horizontal red line indicates the adjusted significance threshold. The black arrows indicate the same genomic region in both panels. (B) Results of the LD analysis for the significantly associated region on chromosome 8 (TRL.8.1). All markers in this peak collapsed into a single LD block. The grayscale coloration indicates the strength of pairwise *r*^2^ values. Significant SNPs are highlighted in yellow, all other markers are suggestive.

The initial set of 1.81M SNPs was reduced to 640,508 SNPs after discarding those with ≥ 30% missing data and/or MAF ≤ 5%. The effective number of independent markers (i.e., tests) was estimated to be 89,009 or ca. 13.9% of the total. Genome-wide association studies were performed for all traits using the filtered SNP set separately for the control and stressed treatments. See Fig 3A for an example of the results for a single trait (TRL), represented as a Manhattan plot for each of the two environments, and S3 Table for a full list of all LS mean phenotypic values per trait/treatment. These analyses resulted in the identification of 21 SNPs on 8 different chromosomes with significant effects on one or more traits in at least one treatment after adjusting for multiple comparisons (i.e., *P* ≤ 5.6E-07 at α = 0.05; for a full list of significant SNPs see S4 Table). These 21 significant SNPs collapsed into 13 unique associated regions. Significant associations were identified for five different traits: root biomass, root biomass allocation, taproot length, stem diameter, and TRL, and ranged in size from a single marker to 186.2 Mbp (Table 2). Note that the inclusion of suggestive markers in the LD analysis extends the size of these associated regions beyond the most distal significant marker (Fig 3B). There were no significant associations identified for stem height, SRL, and TRL allocation. The effect size estimates for these regions ranged from 23.7–39.0%, with no discernible pattern across traits or treatments. Manhattan plots of each trait/treatment combination are in S3 Fig and the LD plots of all significantly associated regions containing more than one marker are in S4 Fig.

**Table 2:** Details for all 13 associated regions. The boundaries of these regions were determined based on observed patterns of LD amongst significant and suggestive markers (see text for details). Marker IDs have been truncated to remove “HanXRQ,” for clarity.

Associated region	Treatment	Chr	Region start marker	Region stop marker	Length of region (Mbp)	Most significant marker	Most significant marker (*P-*value)	Effect of major allele	Effect size (%)	No. genes in region
Root.Biomass.8.1	Water-limited	8	Chr08:80858728	Chr08:91104021	10.25	Chr08:80922968	1.85E-07	-0.002	27.1	118
Root.Biomass.Allocation.10.1	Well-watered	10	Chr10:23215730	Chr10:209437850	186.22	Chr10:129792368	8.38E-08	0.113	39.0	2461
Root.Biomass.Allocation.3.1	Water-limited	3	Chr03:61818236	Chr03:116215926	54.40	Chr03:116035263	1.58E-07	0.077	27.7	705
Stem.Diameter.10.1	Water-limited	10	Chr10:234796538	Chr10:234951032	0.15	Chr10:234938068	5.38E-07	0.195	27.2	2
Stem.Diameter.12.1	Well-watered	12	Chr12:91025435	Chr12:98845333	7.82	Chr12:91065561	3.56E-07	0.250	28.8	92
Stem.Diameter.13.1	Water-limited	13	Chr13:190678838	Chr13:190678838	0.00	Chr13:190678838	4.76E-07	0.244	34.1	3
Stem.Diameter.13.2	Water-limited	13	Chr13:193023389	Chr13:197230300	4.21	Chr13:196651601	8.40E-08	0.202	28.2	133
Stem.Diameter.14.1	Water-limited	14	Chr14:94536231	Chr14:95554794	1.02	Chr14:94676451	1.69E-07	0.221	30.9	6
Stem.Diameter.16.1	Water-limited	16	Chr16:47716478	Chr16:163245935	115.53	Chr16:70632297	1.23E-07	0.185	25.9	1774
Stem.Diameter.8.1	Well-watered	8	Chr08:96933881	Chr08:96933881	0.00	Chr08:96933881	4.57E-07	0.214	24.6	3
Taproot.Length.10.1	Water-limited	10	Chr10:222858781	Chr10:222858781	0.00	Chr10:222858781	3.30E-07	17.406	32.9	3
Taproot.Length.5.1	Water-limited	5	Chr05:1727117	Chr05:1727126	0.00	Chr05:1727126	1.68E-07	12.523	23.7	2
TRL.8.1	Water-limited	8	Chr08:80858728	Chr08:91104021	10.25	Chr08:80922968	2.10E-09	-519.598	26.4	118

In terms of evidence for pleiotropy, only a single genomic region (on chromosome 8; i.e., Chr8) spanning ca. 10.3 Mbp had a significant effect on two different traits: root biomass and TRL; both under stressed conditions. Within this region, the same marker (HanXRQChr08:80922968) was the most significantly associated SNP for both traits, suggesting that variation in these two traits might have a common genetic basis. Interestingly, these were also the only associations for size-related traits for which the major allele had a negative effect on the trait value, indicating that plants carrying the more common allele at this locus have a lower root biomass and shorter TRL under stress as compared to plants carrying the rare allele. There were no instances in which the same chromosomal region had significant effects on one or more traits when comparing across treatments.

As noted in the Materials and Methods, the Bonferroni correction employed herein results in a highly conservative significance threshold that could result in false negatives, even after adjusting for non-independence amongst markers. We thus interrogated the *P*-values of the most significant SNP from each significantly associated region for evidence of possible effect on other traits (whether or not these traits had significant associations of their own), or in the other treatment. The results of this analysis are presented in Table 3. Looking across traits within a treatment, there were 23 instances in which the *P*-value of the focal SNP fell in the top 1% of all *P*-values for one another trait. Similarly, there were eight additional instances in which the *P*-value of the focal SNP fell in the top 5% of all *P*-values for another trait, and six additional instances in which the *P*-value of the focal SNP fell in the top 10% of all *P*-values for another trait. Overall, for 10 of the 13 associated regions, the focal SNP fell in the top 1% of all *P*-values for at least one (and as many as three) other traits, suggesting that these regions might have otherwise undetected effects on multiple traits. For all size-related traits, the effects were always in the same direction (i.e., an increase in the focal trait was accompanied by an increase in the traits for which there is suggestive evidence of an effect). For example, on Chr10 and Chr13, the regions associated with stem diameter under stressed conditions (i.e., Stem.Diameter.10.1 and Stem.Diameter.13.1) had strongly suggestive effects (i.e., top 1%) on taproot length, root biomass, and TRL in the same treatment. In both cases, the major allele produced an increase in stem diameter as well as in all three of the other traits. The only instances in which the sign of the effect changes were for allocation-related traits, which are expressed as a ratio and thus not directly indicative of size.

**Table 3:** Results of analysis for potential pleiotropic effects of all associated regions. Colors indicate the percentile rank of a given marker’s P-value for the alternative trait within treatments or alternative treatment within traits. The 99th, 95th, and 90th percentile are colored dark, medium, and light blue respectively. Blank cells indicate <90th percentile. An X indicates an invalid comparison of a trait to itself while an equals sign (=) denotes same direction of allelic effect between the associated region in the alternative trait within treatments or alternative treatment within traits (e.g., a positive change in the focal trait is associated with a positive change in the other trait), while ≠ denotes a change in the direction of allelic effect (e.g., a positive change in the focal trait is associated with a negative change in the other trait). Marker IDs have been truncated to remove “HanXRQ,” for clarity.

Associated Region						Within treatment								Alternative treatment
Associated region	Treatment	Chr	Most significant marker in region	Most significant marker (*P*-value)	Effect of major allele	Stem Diameter	Stem Height	Taproot Length	Root Biomass	TRL	SRL	Biomass Allocation	TRL Allocation	
Stem.Diameter.8.1	Well-watered	8	Chr08:96933881	4.57E-07	0.214	X		**=**	**=**	**=**				** **
Stem.Diameter.10.1	Water-limited	10	Chr10:234938068	5.38E-07	0.195	X		**=**	**=**	**=**				**=**
Stem.Diameter.12.1	Well-watered	12	Chr12:91065561	3.56E-07	0.25	X	**=**	**=**	**=**	**=**	**=**			** **
Stem.Diameter.13.1	Water-limited	13	Chr13:190678838	4.76E-07	0.244	X		**=**	**=**	**=**				**=**
Stem.Diameter.13.2	Water-limited	13	Chr13:196651601	8.40E-08	0.202	X			**=**	**=**				**=**
Stem.Diameter.14.1	Water-limited	14	Chr14:94676451	1.69E-07	0.221	X			**=**	**=**				**=**
Stem.Diameter.16.1	Water-limited	16	Chr16:70632297	1.23E-07	0.185	X		**=**	**=**	**=**				**=**
Taproot.Length.5.1	Water-limited	5	Chr05:1727126	1.68E-07	12.523	**=**	**=**	X		**=**		**≠**	**≠**	**=**
Taproot.Length.10.1	Water-limited	10	Chr10:222858781	3.30E-07	17.406	** **		X		**=**				**=**
Root.Biomass.8.1	Water-limited	8	Chr08:80922968	1.85E-07	-0.002	** **			X	**=**		**≠**	**≠**	** **
TRL.8.1	Water-limited	8	Chr08:80922968	2.10E-09	-519.598	** **			**=**	X		**≠**	**≠**	**=**
Root.Biomass.Allocation.3.1	Water-limited	3	Chr03:116035263	1.58E-07	0.077	** **					**=**	X	**=**	**=**
Root.Biomass.Allocation.10.1	Well-watered	10	Chr10:129792368	8.38E-08	0.113	** **					**=**	X	**=**	** **

Looking across treatments, there were five instances in which the *P*-value of the focal SNP for a given association in one treatment fell in the top 1% of all *P*-values for that same trait in the other treatment. These associations corresponded to stem diameter, taproot length, and TRL. Similarly, there were three and one additional cases in which the *P*-value of the focal SNP for a given association fell in the top 5% or 10% of the *P*-values for that same trait in the other treatment, respectively. In all cases, the original association was identified under stressed conditions, with a suggestive effect under control conditions. Moreover, in all cases, the focal SNP had the same direction of effect across treatments. For all associated regions, exact pleiotropic percentile values and direction of effects can be found in S5 Table.

### Candidate gene identification

The 13 associated regions contained 5,302 unique genes based on the HanXRQ sunflower genome annotation v1.2 (Table 2). Of these, three relatively large regions on Chr3, Chr10, and Chr16, corresponding to root biomass allocation under stressed and control conditions (i.e., Root.Biomass.Allocation.3.1 [54.4 Mbp in length] and Root.Biomass.Allocation.10.1 [186.2 Mbp in length], respectively) and stem diameter under control conditions (Stem.Diameter.16.1; 115.5 Mbp), contained 705, 2461, and 1774, respectively. The remainder of the associated regions ranged in size from single markers to 10.3 Mbp. Of these ten regions, the number of contained genes varied from 2 to 133, with three single marker associations (HanXRQChr08:96933881 [Stem.Diameter.8.1; control conditions], (HanXRQChr10:222858781 [Taproot.Length.10.1; stressed conditions], and HanXRQChr13:190678838 [Stem.Diameter.13.1; stressed conditions]) falling within genes; the remaining single marker association was located between genes. In all cases, the default buffer of one gene in either direction was included.

Of the 5,302 genes underlying significant associations, 1,511 corresponded to proteins of unknown function or uncharacterized proteins (see S6 Table for a full list of the genes). The three regions containing more than 500 genes were not searched for relevant candidate genes, as the very large number of genes within each of these regions greatly diminishes our ability to identify meaningful candidates for genes underlying the observed trait variation. The remaining regions were found to contain several potentially relevant candidate genes. Under control conditions, for example, two cytochrome P450 genes were found within the stem diameter association on Chr12 (Stem.Diameter.12.1), while the significant marker (HanXRQChr08:96933881) for Stem.Diameter.8.1 was located in a ubiquitin-activating enzyme. Conversely, under stressed conditions, a late embryogenesis abundant (LEA) gene was found within the root biomass and total root length associations on Chr8 (Root.Biomass.8.1 and total root length TRL.8.1). Additionally, a gene corresponding to pyruvate dehydrogenase (E1) was associated with stem diameter on Chr13 (Stem.Diameter.13.2). Finally, the significant SNP underlying taproot length on Chr10 (Taproot.10.1) was found within a MADS-box transcription factor. The potential functional significance of each of these genes is discussed further below.

## Discussion

Taken together, our results reveal the existence of substantial variation in the phenotypic response of sunflower seedlings to osmotic stress (Table 1). Thus, despite the genetic bottleneck that occurred during the domestication of sunflower [59,89,90], cultivated sunflower appears to have retained considerable functional variability. The observed reduction in aboveground growth under osmotic stress (Table 1) was consistent with previous work in sunflower (e.g., [91–93]) and other species (e.g., [94–96]) where osmotic stress was induced using PEG. In partitioning root morphology into upper vs. lower root sections, we observed a shift from higher TRL allocation (i.e., more lateral root growth near the soil surface) under control conditions towards reduced TRL allocation values (i.e., increased TRL deeper in the soil) under stress (Fig 1). This shift toward decreased TRL allocation, coupled with an increase in SRL and taproot length under osmotic stress drove the separation of treatments across trait space (Fig 2B), and suggests that sunflower seedlings are attempting to avoid water limitation by accessing deeper water sources (e.g., [14,15,29,97,98]).

The 13 significantly associated regions (corresponding to 5 traits) that were identified in this study exhibited substantial variation in physical size, ranging from a single marker to 186.2 Mbp in length (Table 2). This pattern is consistent with an uneven distribution of LD across the genome, as has previously been documented in cultivated sunflower (e.g., [60,89,99,100]). Unsurprisingly, our largest regions (Root.Biomass.Allocation.3.1, Root.Biomass.Allocation.10.1, and Stem.Diameter.16.1) correspond to chromosomal regions with known islands of elevated LD [60]. For example, the largest association that we identified falls within a well-studied region of Chr10 that has an interesting evolutionary history. This region is known to harbor a large introgression from wild sunflower that is responsible for the reintroduction of recessive branching into one of the two heterotic groups of sunflower (i.e., the male or restorer [RHA] lines) resulting in indeterminate growth and a lengthy period of pollen shedding that is important for hybrid seed production [101–104].

Interestingly, for all but one of the associations underlying size-related traits, the major allele resulted in increased plant growth, which suggests that selection for increased vigor during the evolution of cultivated sunflower may be responsible for the relatively high frequency of these alleles (Table 2). The sole outlier from this pattern was the region of Chr8 that associated with both root biomass and TRL under osmotic stress; for both of these traits, the common allele resulted in reduced root biomass and length. The rare allele at this locus could thus be a source of useful variation for efforts aimed at improving root growth, particularly under water-limited conditions. In terms of estimated effect sizes, there was relatively little variation across associated regions, with magnitudes of effect ranging from 23.7–39.0%, and no clear pattern based on type of trait or treatment in which the association was detected. Regardless, these results add to what is known about the genetic basis of growth-related traits in sunflower [8,92,105] and other crop species (e.g., [8,32,33,40,98,106]).

Taken at face value, our results suggest a paucity of pleiotropic effects, with all but one genomic region influencing just a single trait (Table 2). Moreover, the lack of significant associations for any given trait across both treatments suggests that environmental-specificity is the rule when it comes to the genetic basis of the observed trait variation. Digging deeper, however, it appears that these patterns may be an oversimplification, likely arising as a byproduct of the highly conservative significance threshold employed herein (see also [107,108]). Indeed, when looking at the most significant marker in a given region, we see substantial evidence of possible effects across traits (Table 3), which is supported through significant trait-trait correlations in the phenotypic data (Fig 2A). Moreover, these effects are not restricted to combinations of aboveground vs. belowground traits. Rather, there are numerous instances in which regions influencing stem diameter (our primary aboveground trait) appear to also influence belowground trait variation, and vice versa. In this context, it is worth noting that there do not appear to be any clear instances of genetic tradeoffs between aboveground and belowground performance. Rather, it appears that alleles increasing aboveground growth have a similar effect on belowground traits, and vice versa. Indeed, we see positive trait correlations between our aboveground traits and taproot length, root biomass, and TRL in both treatments (Fig 2A), further indicating that greater aboveground growth is associated with greater belowground growth. This finding has important practical implications as, for example, selection for alleles conferring deeper rooting in a given treatment will likely not come at the cost of aboveground growth.

In terms of cross-treatment comparisons, our results were similar to the cross-trait comparisons. While we only detected particular associations in one treatment or the other, suggesting the possibility of pervasive genotype-by-treatment interactions, nine of thirteen associated regions had evidence supporting a possible effect in the alternative treatment (Table 3). While this general approach to investigating the environment-dependence of genetic effects (i.e., the independent analysis of traits under varying environmental conditions) is quite common (e.g., [39,109,110]), our results highlight the dangers of simply comparing significant genomic regions between treatments (Fig 3A). Interestingly, in all cases, the direction of the phenotypic effects of the major vs. minor allele is unchanged across treatments. This breaks from the traditional view that relatively high performance under stress comes at a cost of reduced performance under benign conditions (e.g., [3,24,53]). While we cannot rule out the possibility of genotype-by-treatment interaction in terms of the magnitudes of effects, our results indicate that the directionality of allelic effects tends to be consistent across conditions. Thus, as others have argued, alleles conferring high performance under ideal conditions are likely to confer high performance under stressful conditions (e.g., [53–55]). Of course, not all associated regions showed evidence of environment-independence. In fact, as noted above, four of the associated regions that we identified had no evidence of an effect in the alternate treatment (i.e., the focal SNP for each of these regions was not even in the top 10% of *P*-values in the alternate treatment), indicating that the associations we detected included a mix of environmentally dependent and independent genetic effects.

Given the large size of some of our associated regions, along with the large number of genes contained within such regions (Table 2 and S6 Table), the identification of promising candidate genes is a challenging endeavor. Moreover, due to the arguably pleiotropic nature of most of these associated regions, clear search criteria for candidate genes are difficult to formulate. In broad terms, one might expect to identify genes corresponding to some fundamental aspect of plant growth or development. An important caveat here is that our list of candidate genes was derived from the reference sunflower genome, and it is now well known that there is pervasive variation in gene content in plant genomes e.g.,[111]. As such, it is possible that one or more of the genes ultimately responsible for the observed trait variation is absent from the reference genome. It must also be kept in mind that, while genic sequences tend to garner the most interest when it comes to inferring the cause of phenotypic variation, there is increasing appreciation for the possible functional significance of intergenic variants, particularly via their potential regulatory effects (e.g., [112–118]). Our approach to candidate gene identification would obviously miss such non-genic variants.

The above caveats notwithstanding, it is worth noting that under control conditions, two cytochrome P450 genes were found to be associated with stem diameter on Chr12 (Stem.Diameter.12.1; S6 Table). Cytochromes are widespread across the tree of life, particularly in plants [119], and this enzyme superfamily has been linked to various aspects of plant metabolism, synthesis of primary and secondary metabolites, polymerization of lignin, and the formation of complex anatomical structures [119,120]. Additionally, the single marker underlying Stem.Diameter.8.1 was located within a ubiquitin-activating enzyme (i.e., ubiquitin E1; S6 Table). The ubiquitin pathway is involved in the regulation of plant growth and development through the degradation of *Aux/IAA* repressors, thereby promoting auxin-responsive genes [121–123]. Interestingly, while this pathway has been linked to abiotic stress response via the degradation of gene repressors or harmful stress-induced byproducts (e.g., [124,125]), this marker did not show even suggestive evidence of an effect in the stressed environment.

Under stressed conditions, a pyruvate dehydrogenase E1, which was identified out of 133 genes in the region corresponding to Stem.Diameter.13.2, was directly adjacent to the most significant SNP underlying that association. This enzyme is the rate-limiting step for the whole pyruvate dehydrogenase complex, which links carbon output from glycolysis to the Krebs cycle, thereby influencing energy metabolism [126]. We also found a MADS-box transcription factor on Chr10 that contained the only significant marker underlying Taproot.10.1 (Table 2; S6 Table). Members of the MADS-box gene family influence a variety of processes, with many being linked to reproductive development; however, there is evidence that members of this gene family are also involved in vegetative development, particularly root elongation, in numerous plant species [127–131]. Given the close proximity of these genes to the most significant SNPs associated with each trait, they are rather promising candidates. Moreover, the fact that these sorts of genes are known to influence multiple aspects of plant growth and development is consistent with the potentially pleiotropic effects of these regions. Finally, we identified a late embryogenesis abundant (LEA) gene within the region on Chr8 that influences both root biomass and TRL under stressed conditions (i.e., Root.Biomass.8.1 and TRL.8.1). This protein family has a strong association with abiotic stress responses, particularly related to dehydration stress (e.g., [132,133]) Indeed, it is involved in multiple relevant processes, including combating stress-derived ROS responses and maintaining membrane stability during periods of water loss [132,134–136].

## Conclusions

Our results have revealed that sunflower harbors substantial variation for seedling root morphology and growth traits under both well-watered and water-limited conditions. Osmotic stress resulted in decreased aboveground growth accompanied by a shift towards deeper rooting, with significant line-by-treatment interactions across our diversity panel. While this work provides superficial support for pervasive trait- and treatment-specific genetic effects, closer inspection of the data has revealed evidence of pleiotropic effects across multiple traits, as well as numerous examples of environmentally independent genetic effects. Overall, our results suggest the existence of little in the way of tradeoffs between aboveground and belowground growth characteristics, and further suggest that the majority of alleles have consistent effects across treatments. As such, it appears that selection for alleles conferring deeper rooting in a given treatment will likely not come at the cost of aboveground growth, and that selection for high performance in one environment may result in improved performance under other conditions. We also identified candidate genes underlying these associated regions, some of which correspond to genes involved in various aspects of plant growth/development and response to osmotic stress.

## Supporting information

S1 Fig Population structure of the SAM population. Population structure is illustrated using the first four principal components based on the genome-wide collection of SNPs.
(PDF)

S2 Fig Kinship of the SAM population. Relatedness values were calculated using all SNPs in EMMAX. Warmer (i.e., redder) colors indicate higher relatedness. Along the left border are genotype classification information for heterotic group and market type.
(TIF)

S3 Fig Manhattan plots for all measured traits. Colors alternate by chromosome, dots correspond to SNPs, and the horizontal red line indicates the adjusted significance threshold.
(PDF)

S4 Fig Linkage disequilibrium (LD) plots for all associated regions that contain multiple significant markers. Colors along the diagonal indicate similar groups of markers based on *r*^2^. Plots separated per chromosome, per trait, per treatment. As described in the methods we used LDSelect to determine the boundaries of the region showing strong LD with significant SNPs. Significant SNPs are marked in yellow, all others are suggestive.
(PDF)

S1 Table Accession information for the SAM population. (XLSX)

S2 Table Summary of trait-trait correlations within and across treatments. (XLSX)

S3 Table Summary of phenotypic values for all mapped traits. (XLSX)

S4 Table Full list of significant SNPs by associated region. (XLSX)

S5 Table Full list of putative pleiotropic effects for associated regions within and across treatments. (XLSX)

S6 Table Full list of genes underlying associated regions. (XLSX)
