# Supplementary figures and images for: Multiple genomic regions influence root morphology and seedling growth in cultivated sunflower (Helianthus annuus L.) under well-watered and water-limited conditions
Source: PLoS One. 2018 Sep 20;13(9):e0204279. doi: 10.1371/journal.pone.0204279 (PMC6147562; doi:10.1371/journal.pone.0204279)

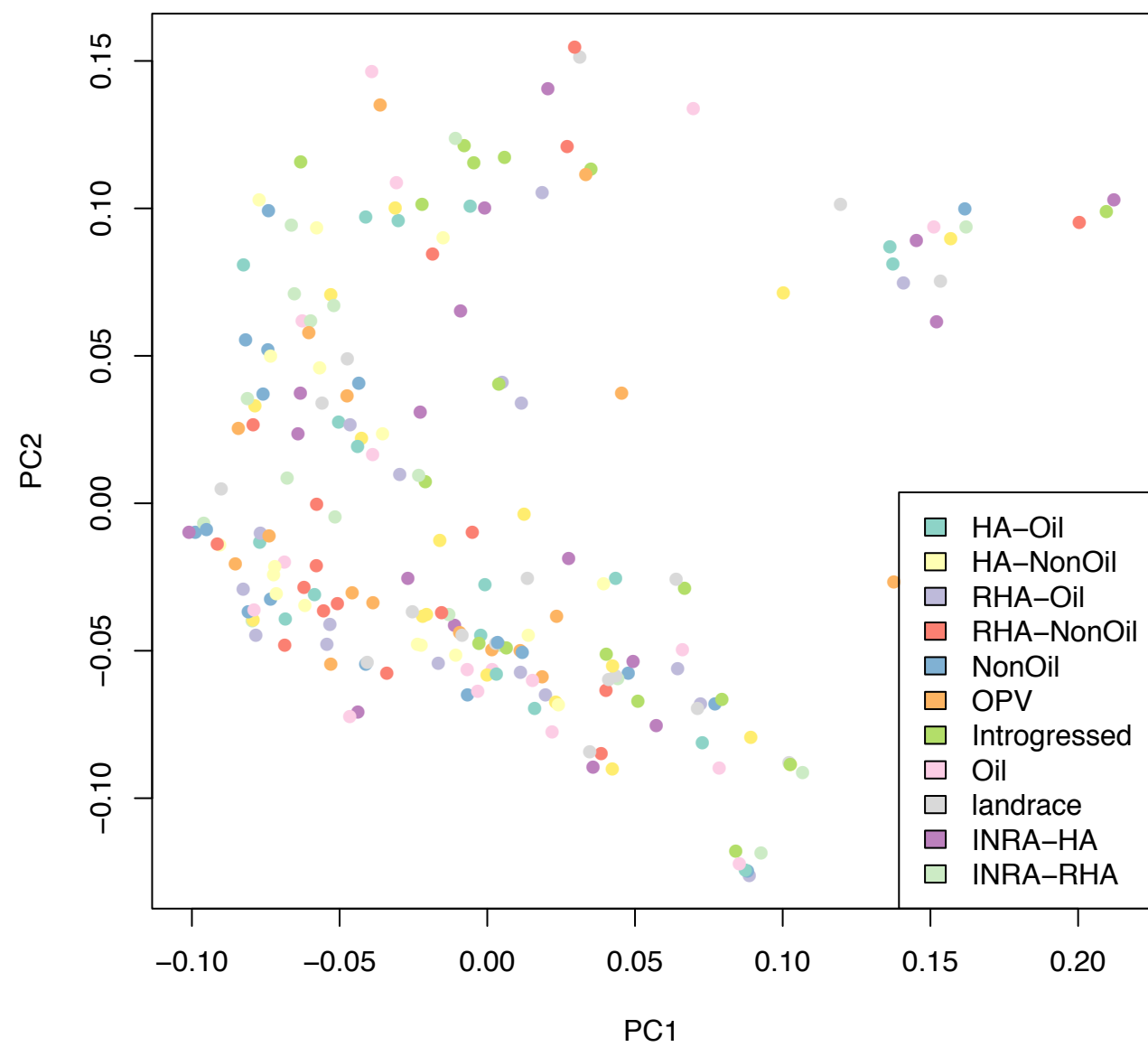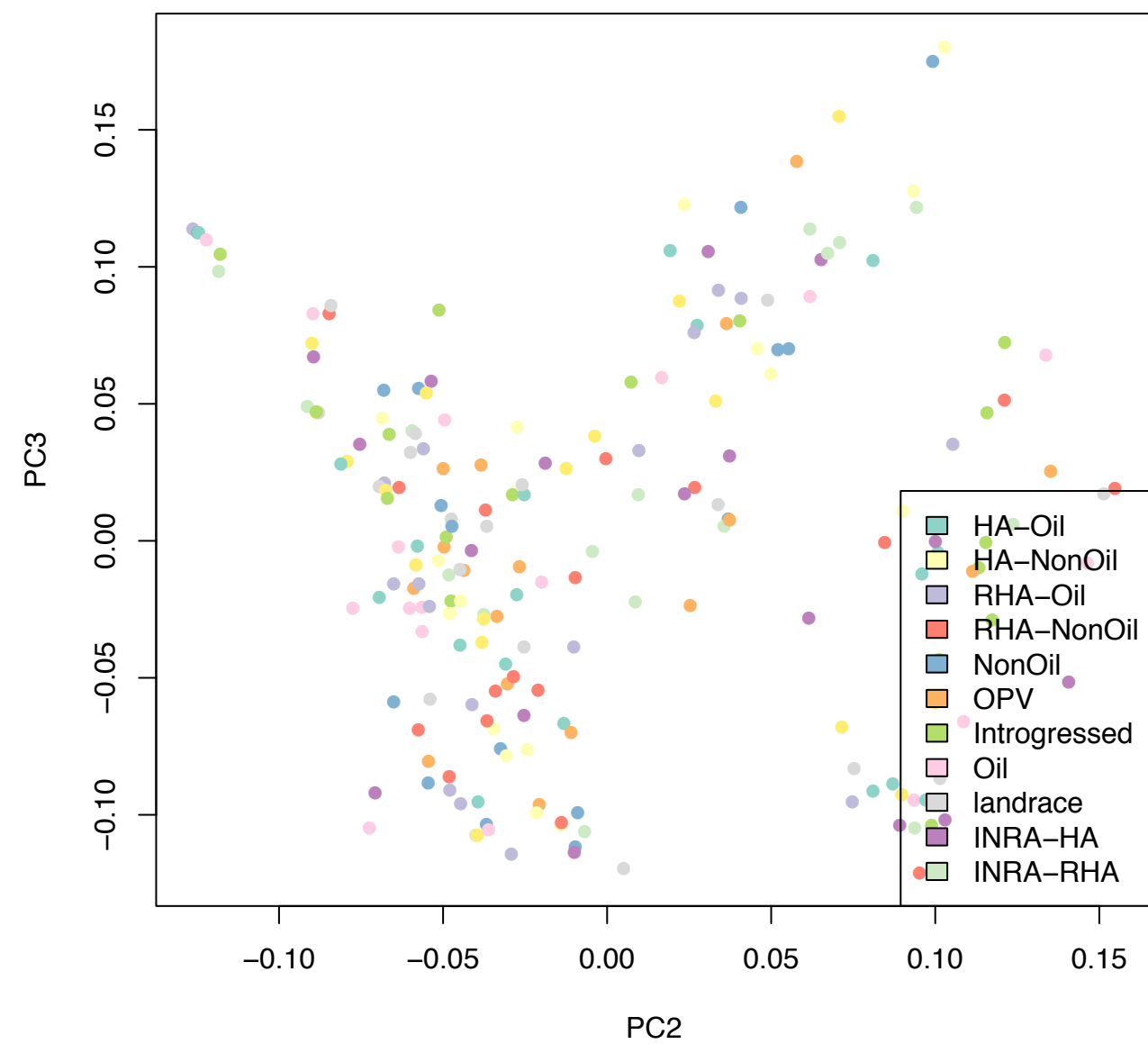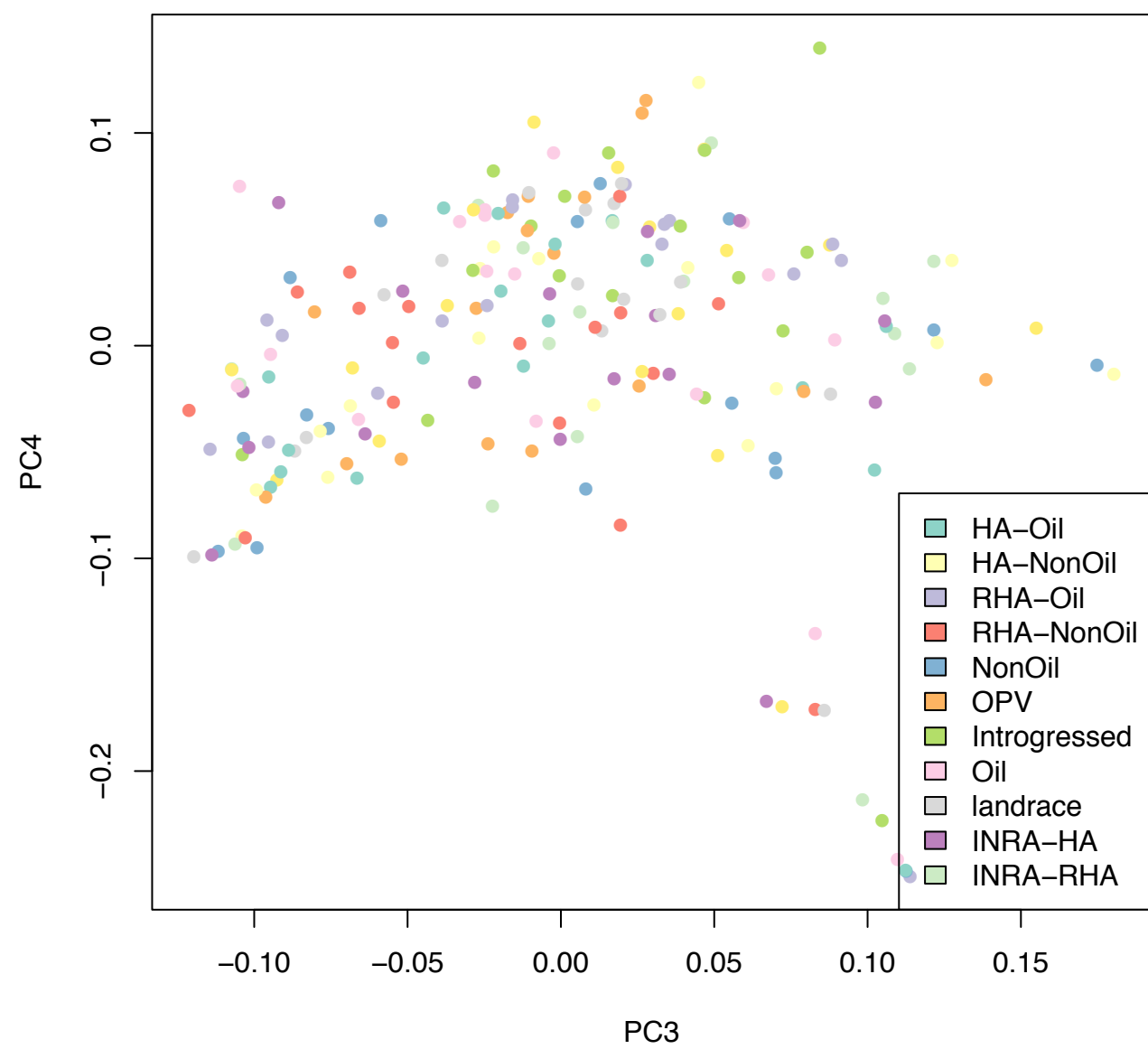

Supplement: S1 Fig — Population structure is illustrated using the first four principal components based on the genome-wide collection of SNPs. (PDF) [file pone.0204279.s001.pdf]

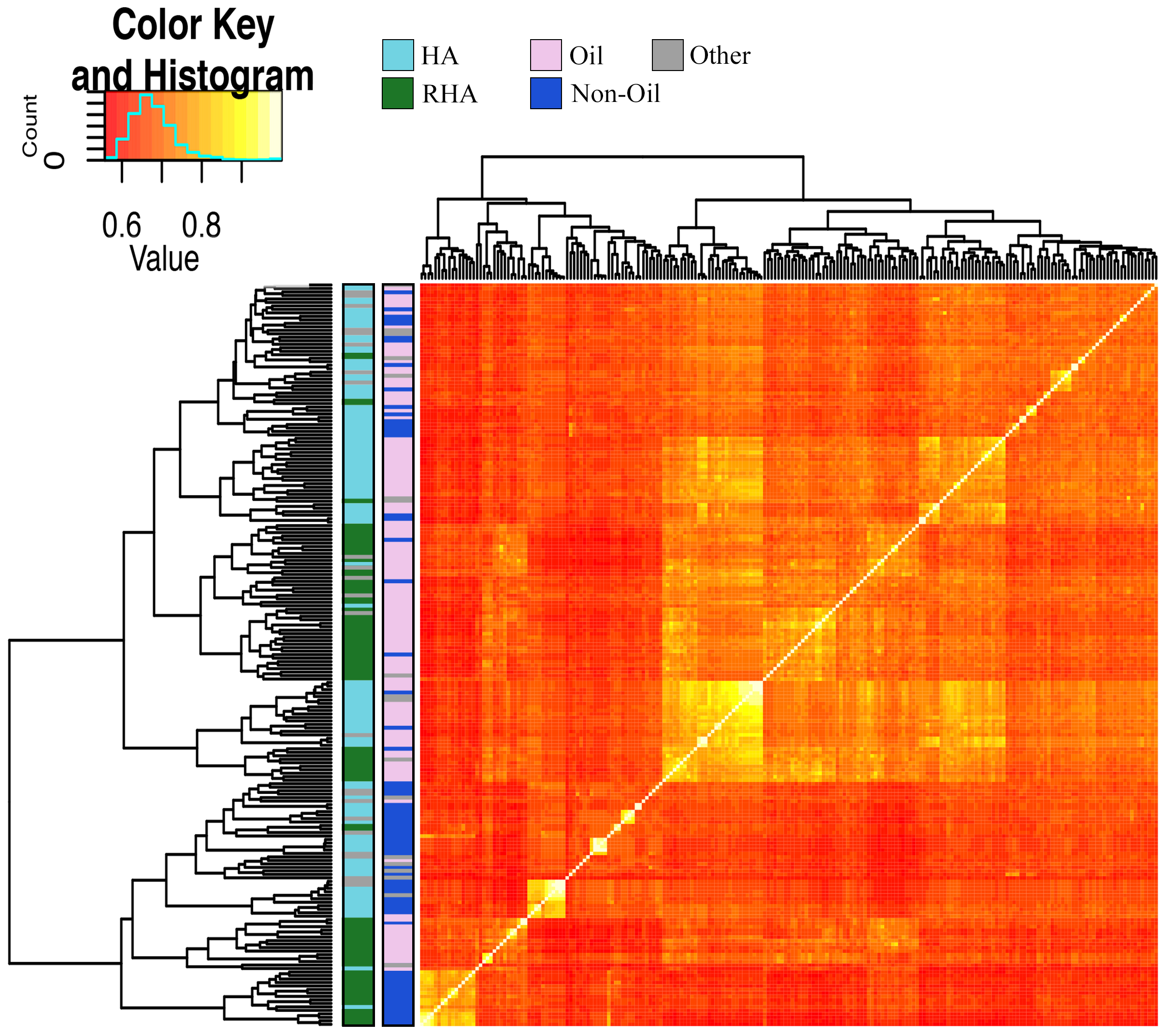

Supplement: S2 Fig — Relatedness values were calculated using all SNPs in EMMAX. Warmer (i.e., redder) colors indicate higher relatedness. Along the left border are genotype classification information for heterotic group and market type. (TIF) [file pone.0204279.s002.tif]
