# Supplementary material for: Multiple genomic regions influence root morphology and seedling growth in cultivated sunflower (Helianthus annuus L.) under well-watered and water-limited conditions
Source: PLoS One. 2018 Sep 20;13(9):e0204279. doi: 10.1371/journal.pone.0204279 (PMC6147562; doi:10.1371/journal.pone.0204279)
Supplement: S3 Fig — Colors alternate by chromosome, dots correspond to SNPs, and the horizontal red line indicates the adjusted significance threshold. (PDF) [file pone.0204279.s003.pdf]

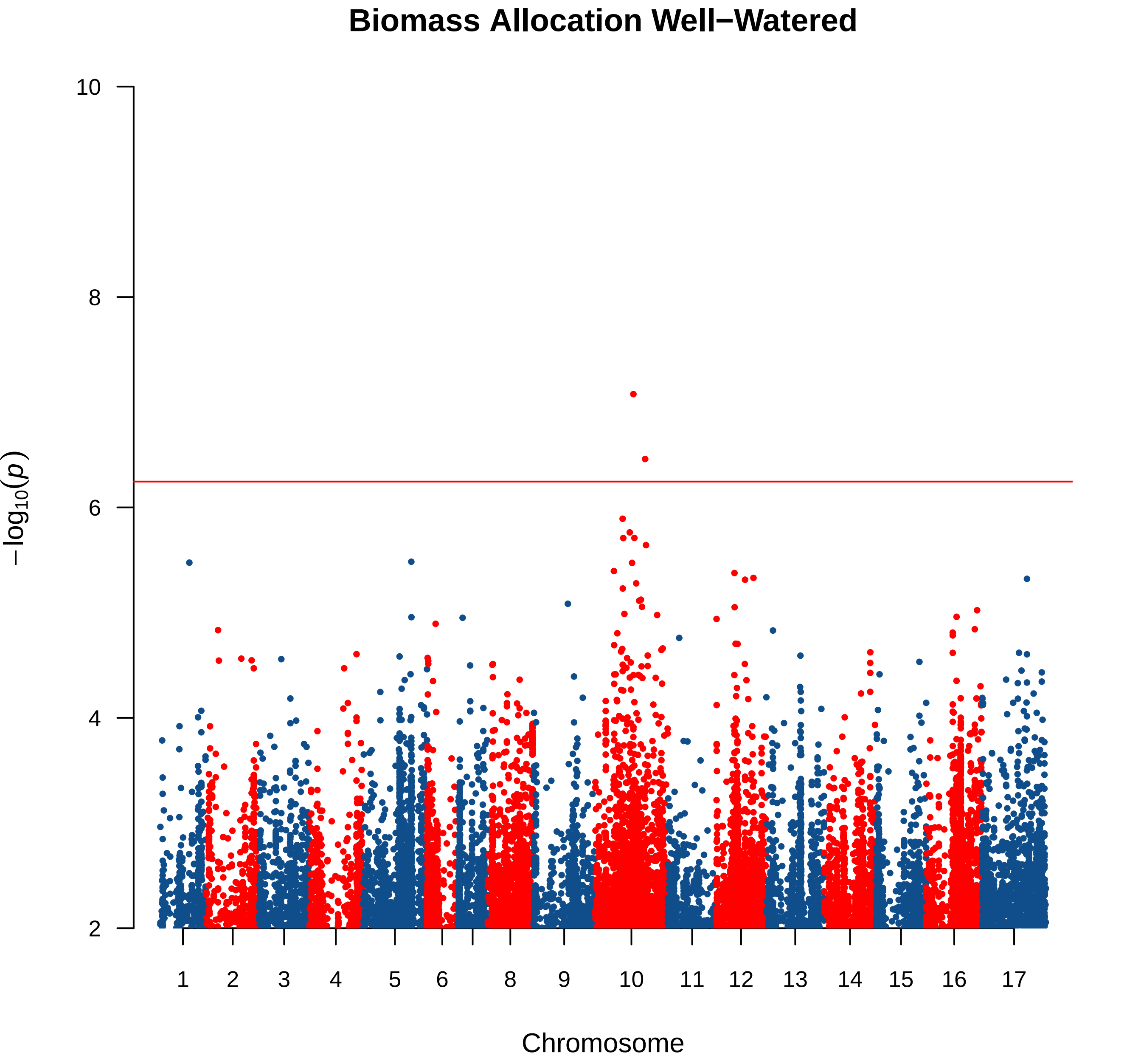
Biomass Allocation Well−Watered
10
8
6
4
2
−log10(p)
1
2
3
4
5
6
8
9
10
11
12
13
14
15
16
17
Chromosome

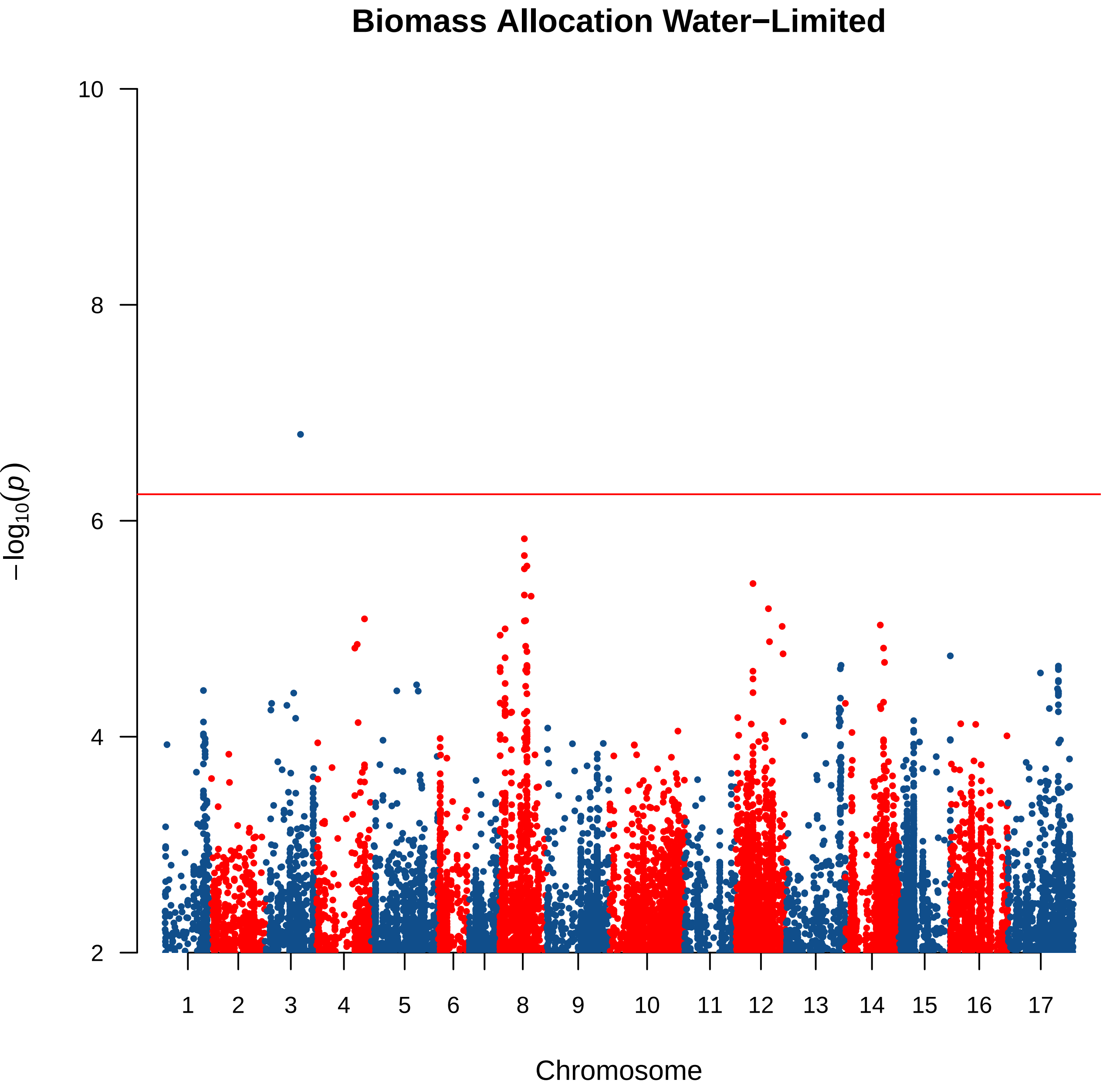

Biomass Allocation Water−Limited
10
8
6
4
2
−log10(p)
1
2
3
4
5
6
8
9
10
11
12
13
14
15
16
17
Chromosome

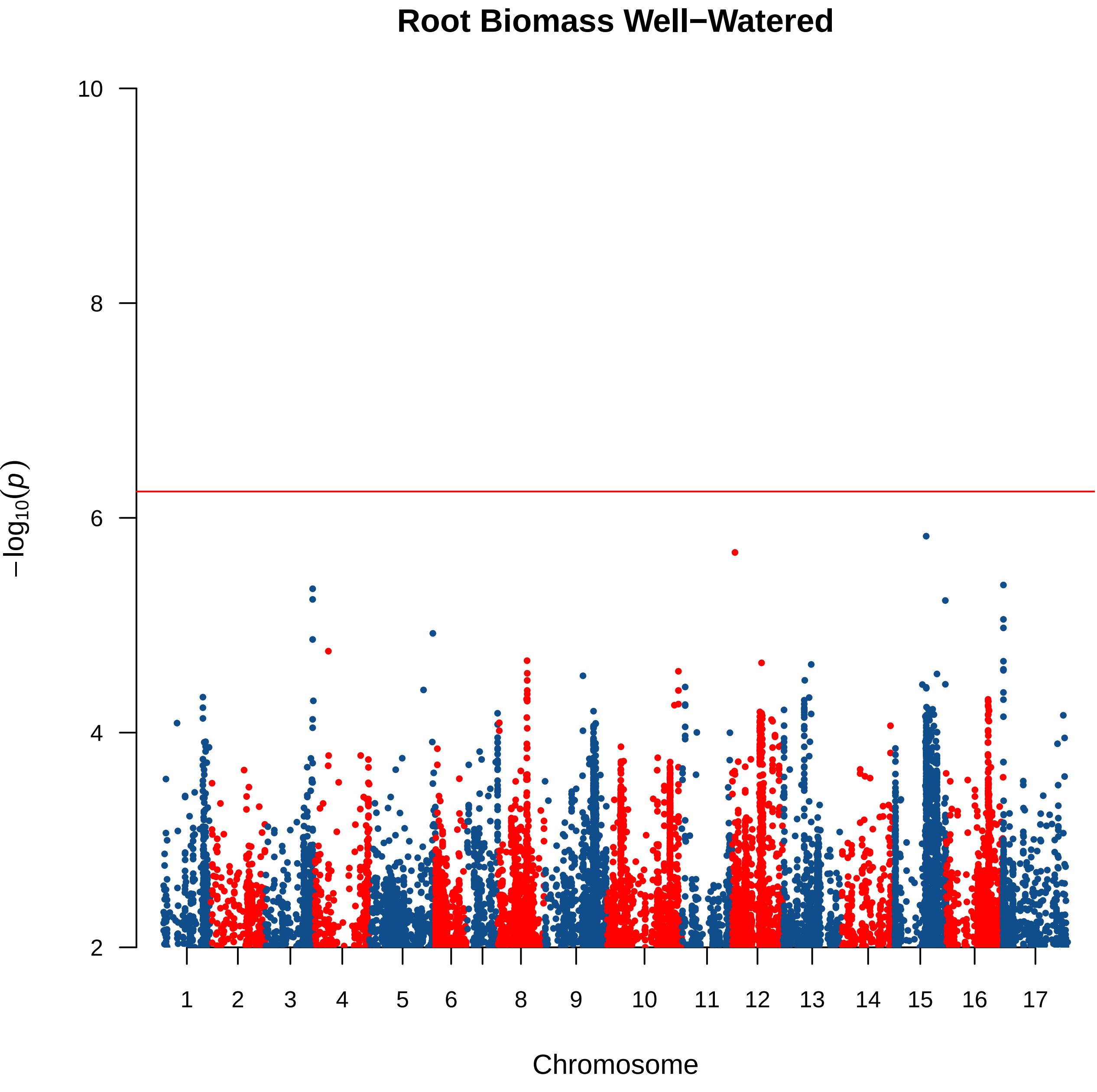

Root Biomass Well−Watered
10
8
6
4
2
−log10(p)
1
2
3
4
5
6
8
9
10
11
12
13
14
15
16
17
Chromosome

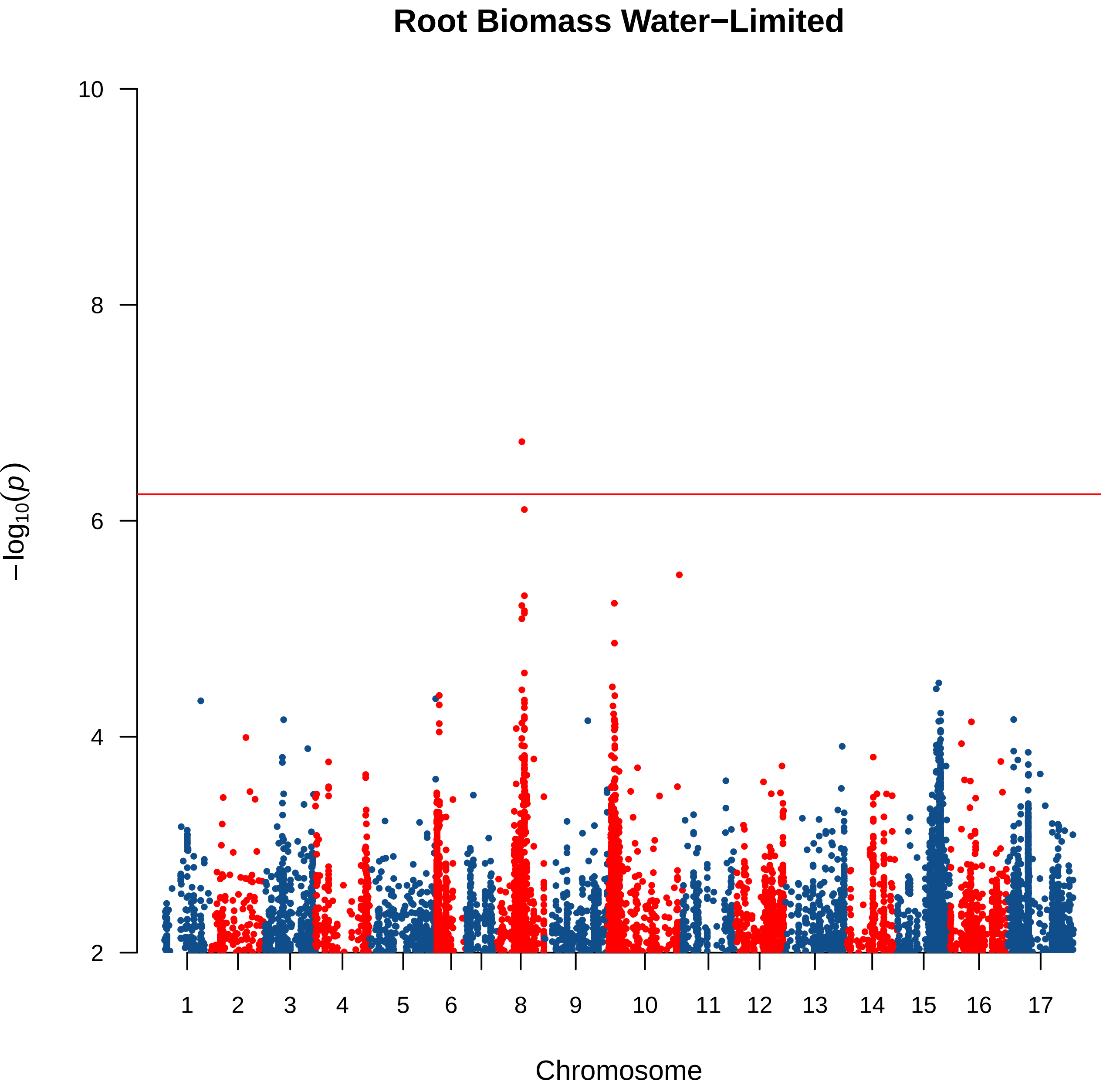
Root Biomass Water−Limited
10
8
6
4
2
−log10(p)
1
2
3
4
5
6
8
9
10
11
12
13
14
15
16
17
Chromosome

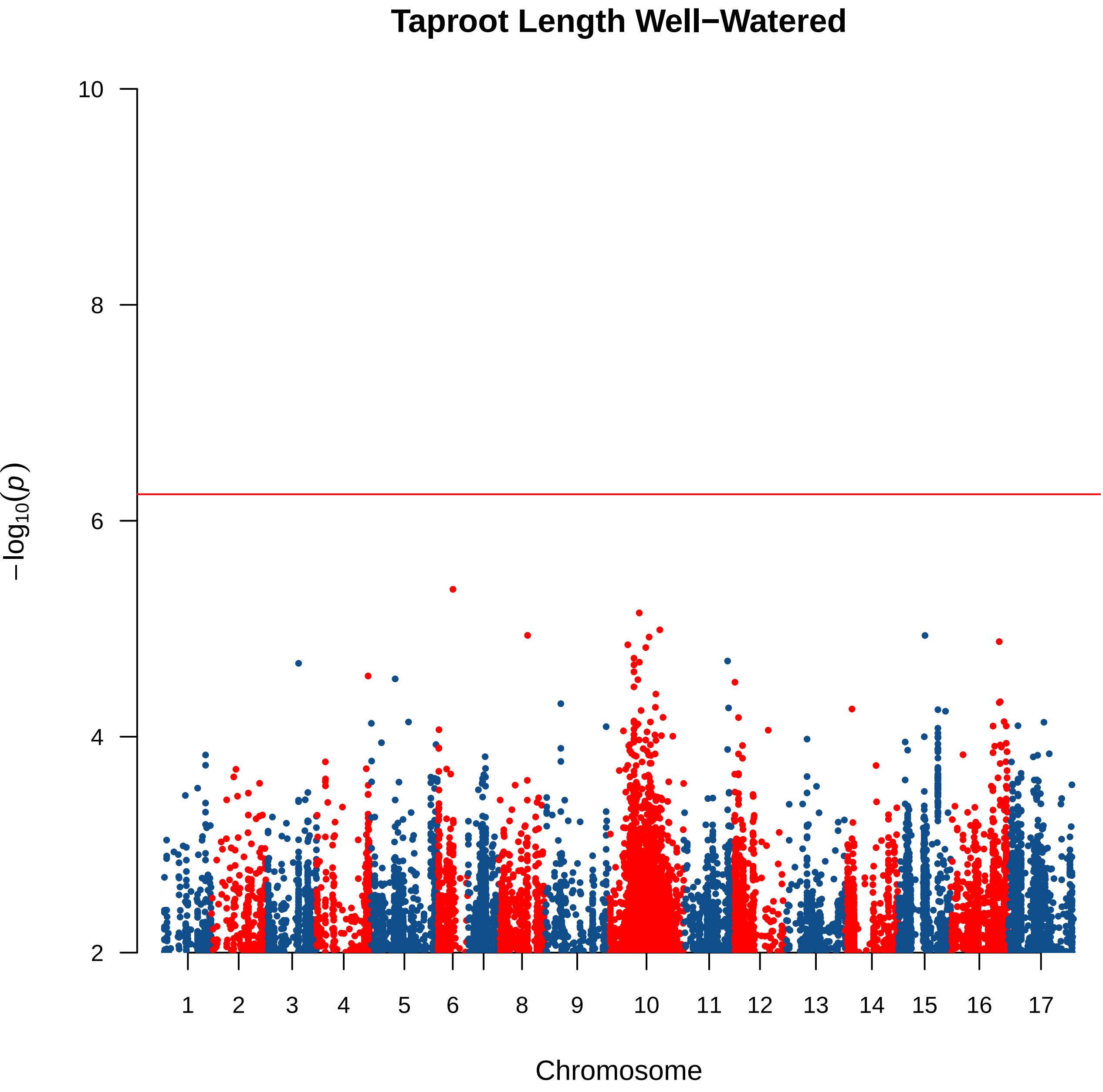

Taproot Length Well−Watered
10
8
6
4
2
−log10(p)
1
2
3
4
5
6
8
9
10
11
12
13
14
15
16
17
Chromosome

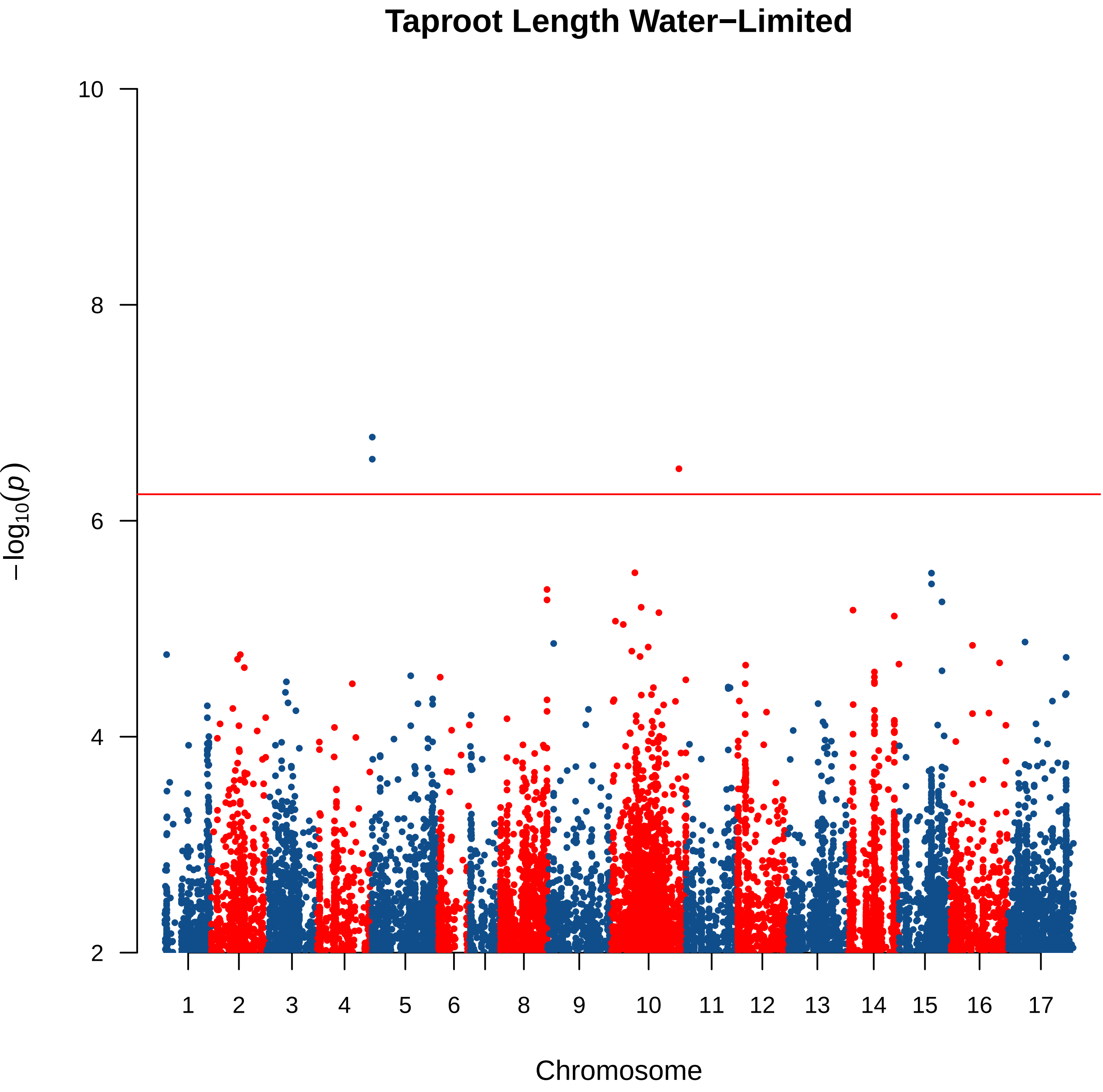
Taproot Length Water−Limited
10
8
6
4
2
−log10(p)
1
2
3
4
5
6
8
9
10
11
12
13
14
15
16
17
Chromosome

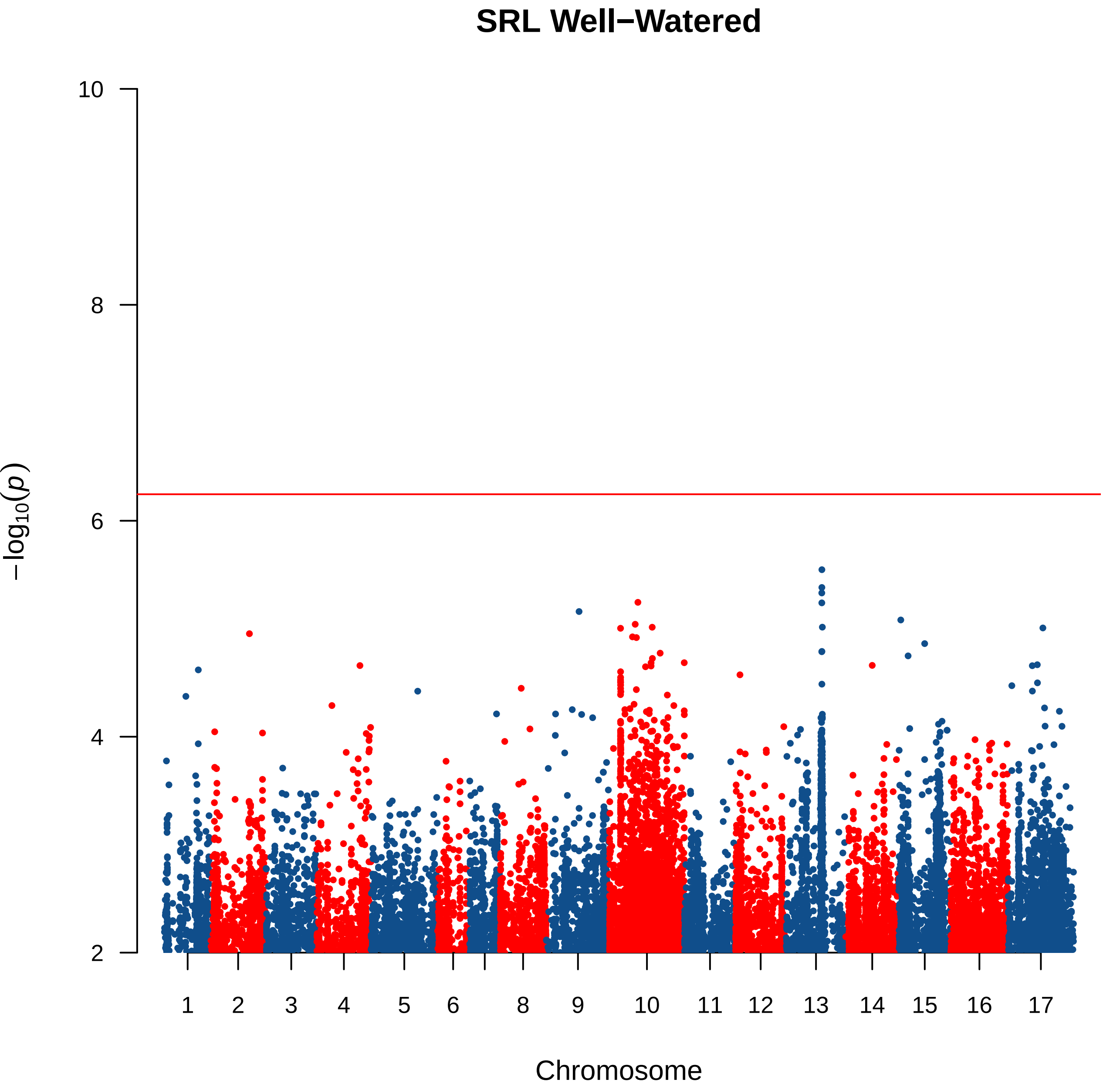
SRL Well−Watered
10
8
6
4
2
−log10(p)
1
2
3
4
5
6
8
9
10
11
12
13
14
15
16
17
Chromosome

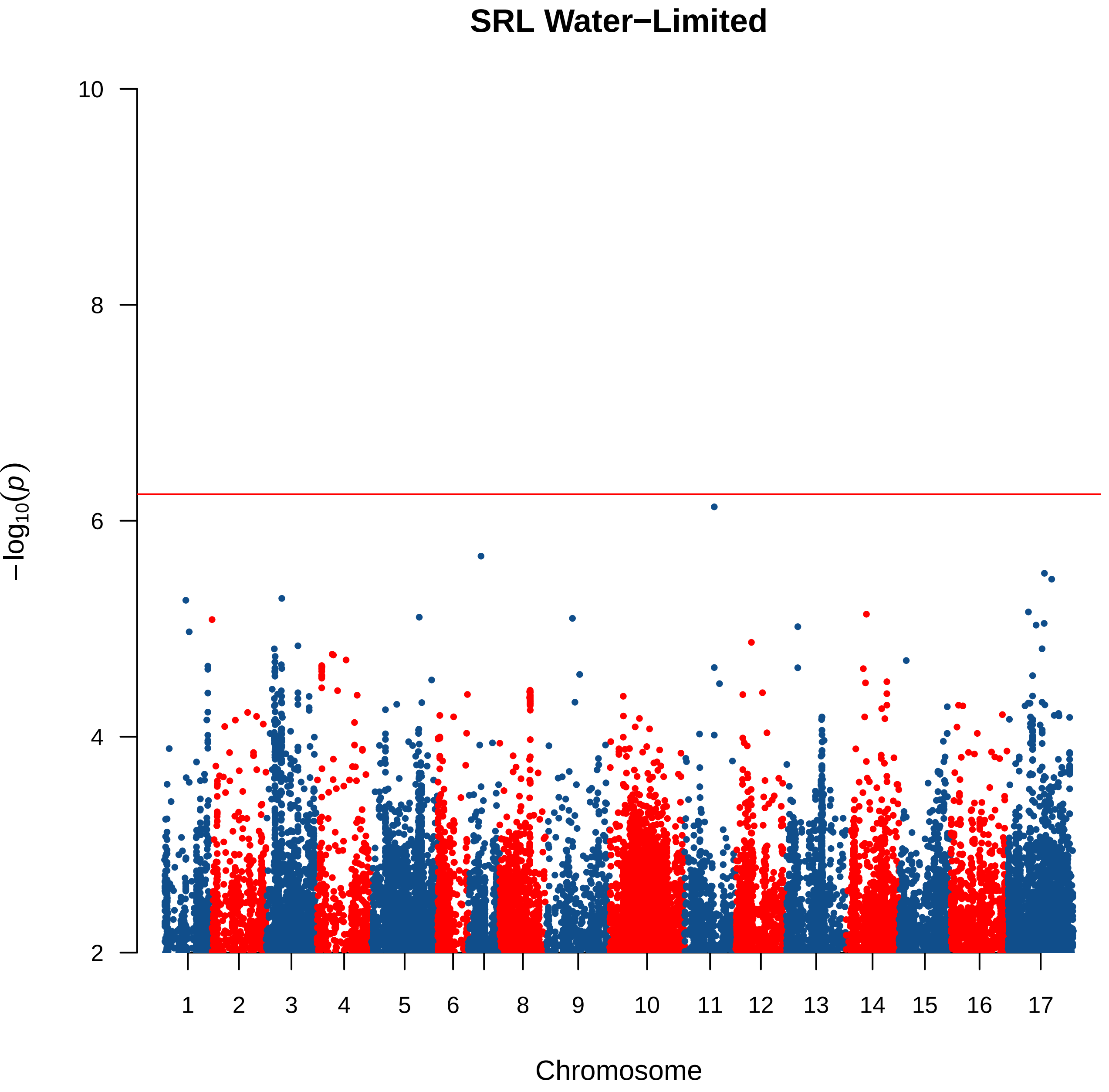

SRL Water−Limited
10
8
6
4
2
−log10(p)
1
2
3
4
5
6
8
9
10
11
12
13
14
15
16
17
Chromosome

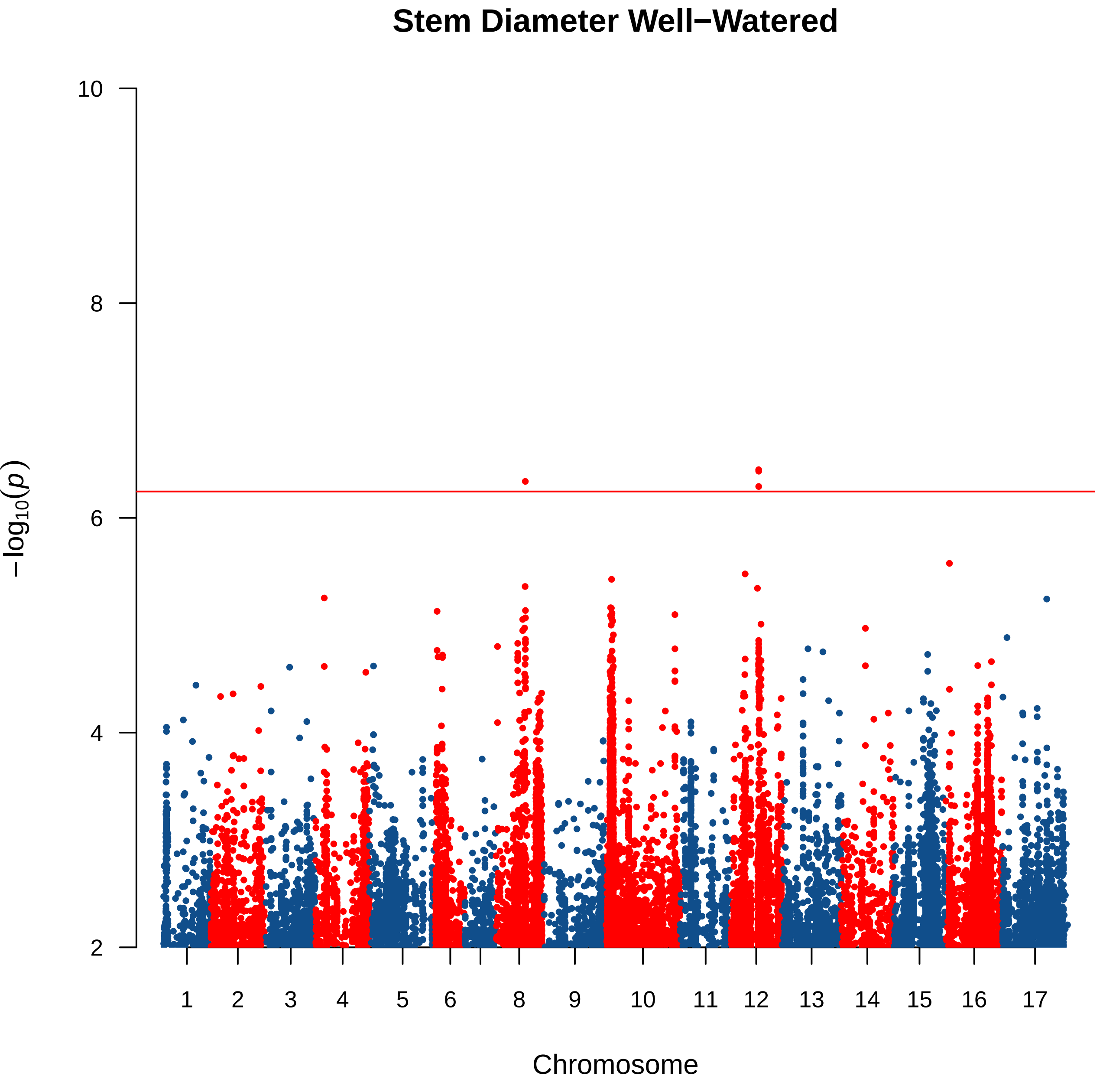
Stem Diameter Well−Watered
10
8
6
4
2
−log10(p)
1
2
3
4
5
6
8
9
10
11
12
13
14
15
16
17
Chromosome

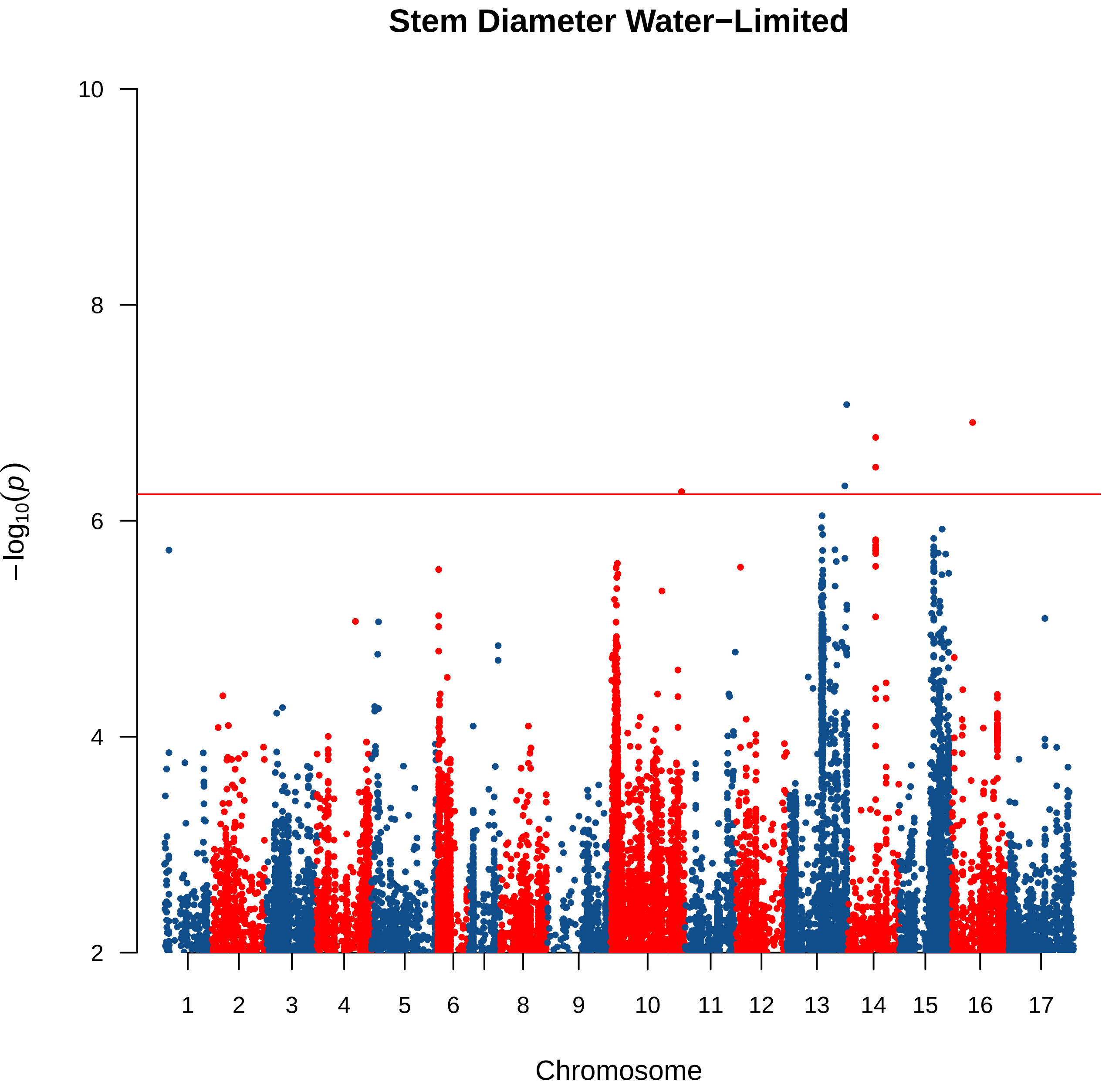
Stem Diameter Water−Limited
10
8
6
4
2
−log10(p)
1
2
3
4
5
6
8
9
10
11
12
13
14
15
16
17
Chromosome

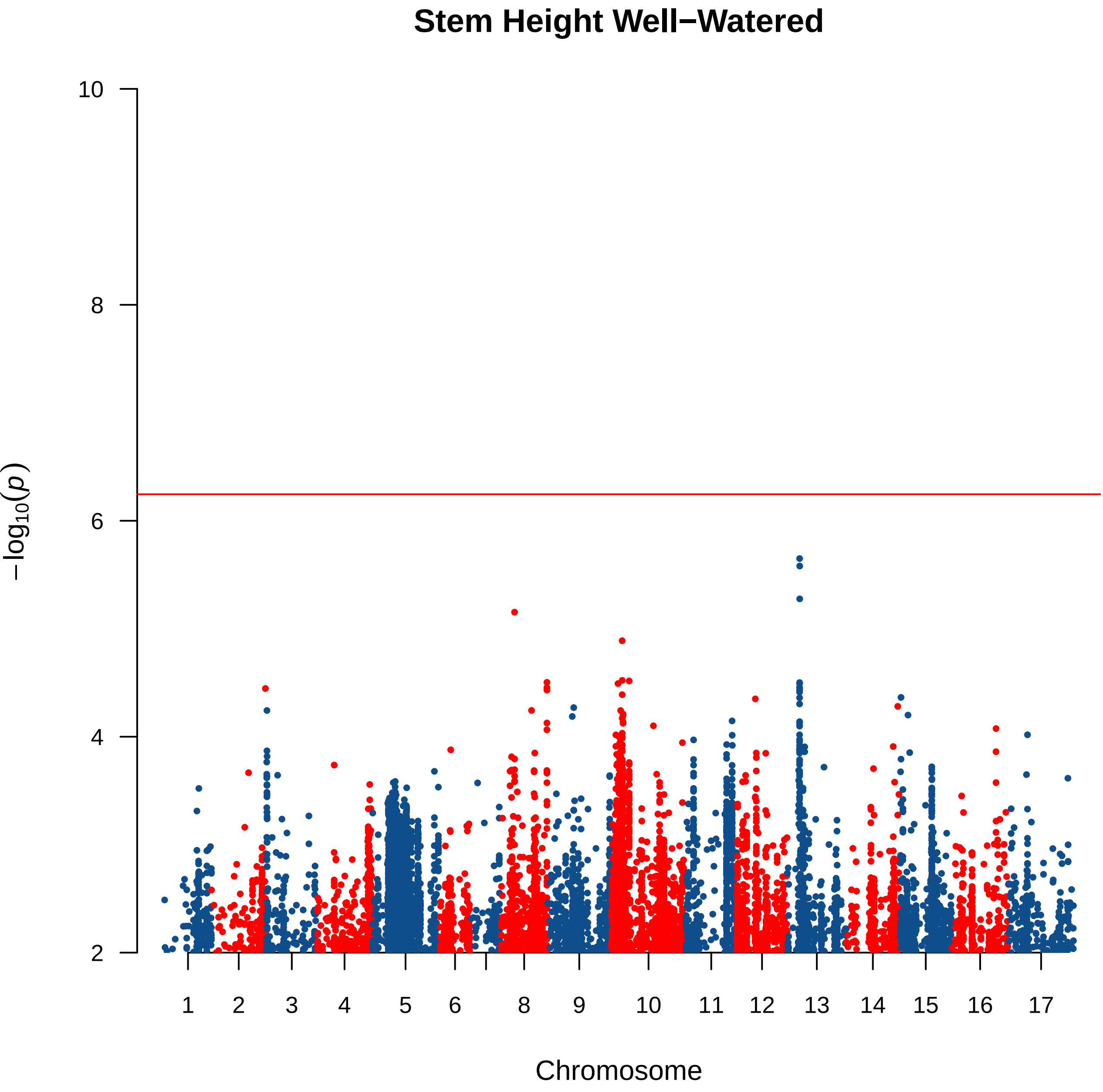
Stem Height Well−Watered
10
8
6
4
2
−log10(p)
1
2
3
4
5
6
8
9
10
11
12
13
14
15
16
17
Chromosome

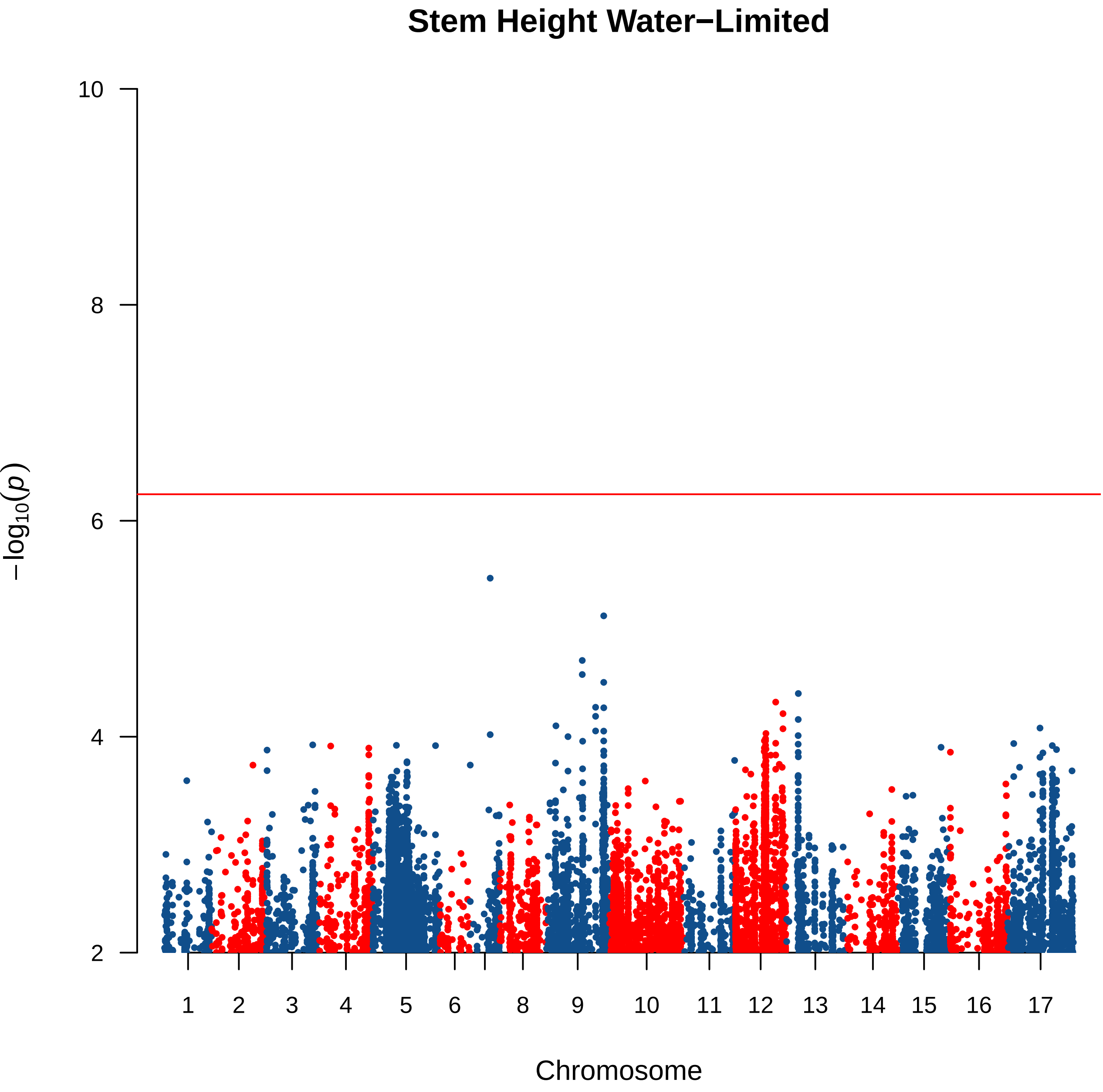
Stem Height Water−Limited
10
8
6
4
2
−log10(p)
1
2
3
4
5
6
8
9
10
11
12
13
14
15
16
17
Chromosome

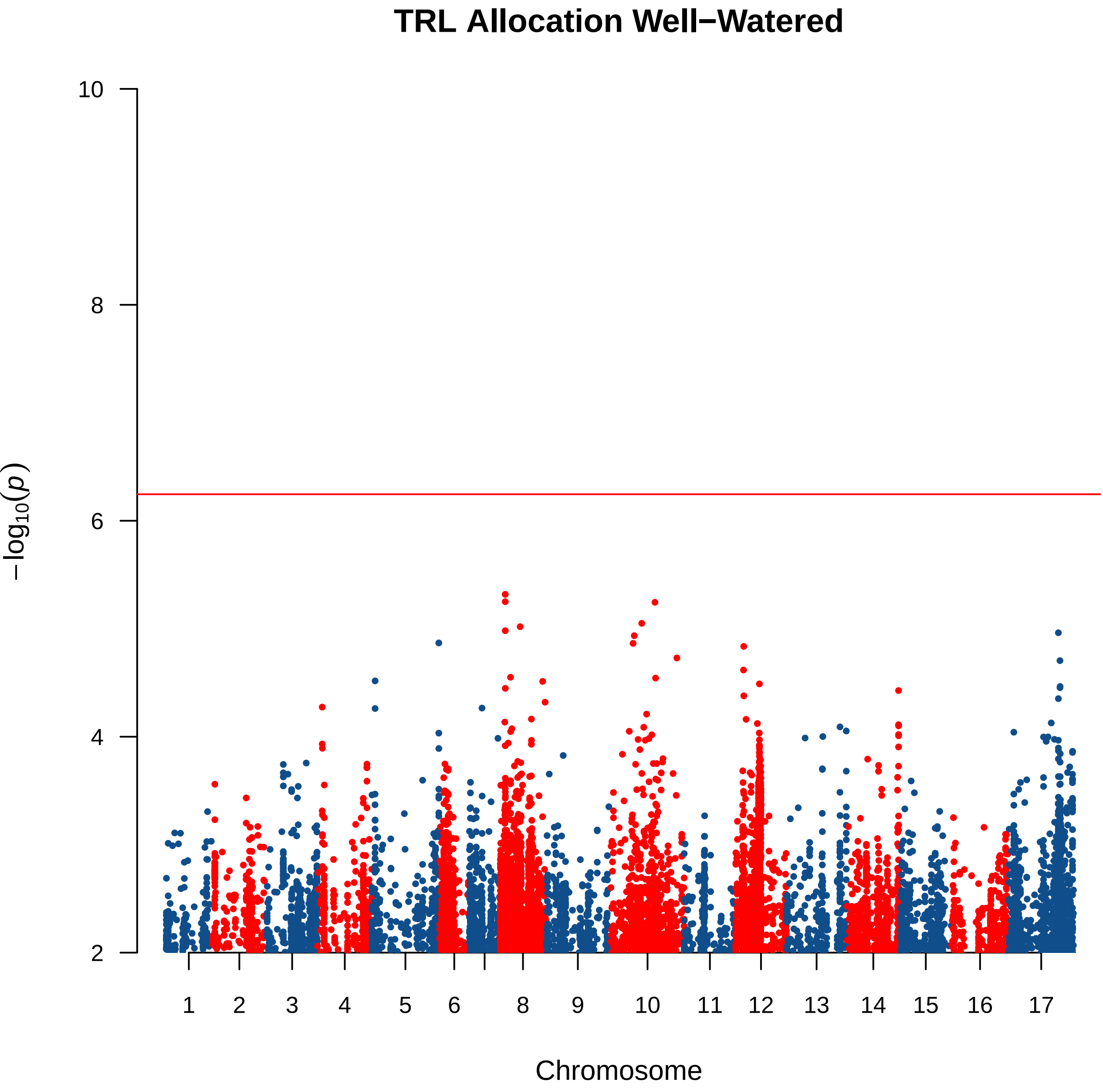

TRL Allocation Well−Watered
10
8
6
4
2
−log10(p)
1
2
3
4
5
6
8
9
10
11
12
13
14
15
16
17
Chromosome

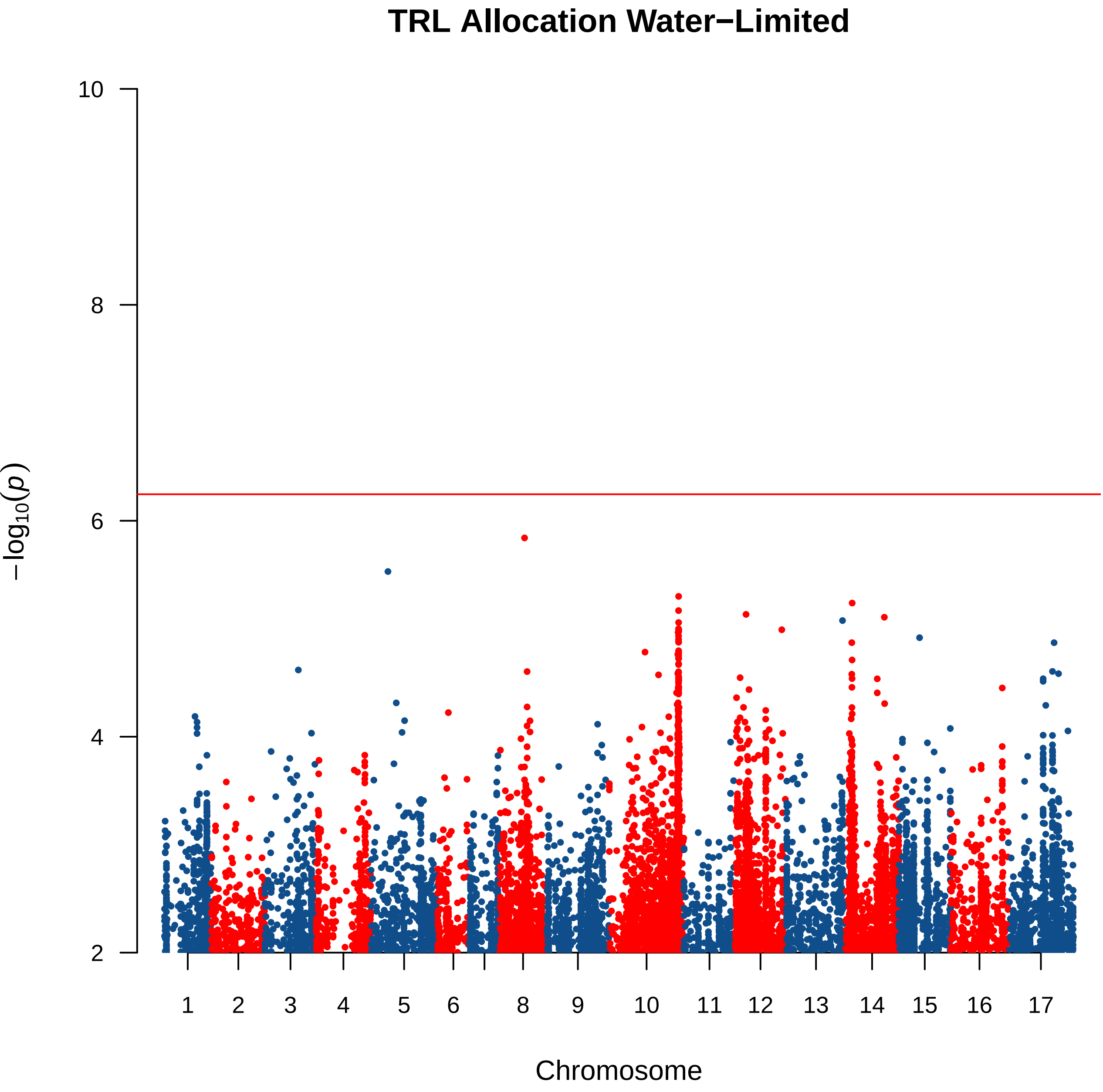
TRL Allocation Water−Limited
10
8
6
4
2
−log10(p)
1
2
3
4
5
6
8
9
10
11
12
13
14
15
16
17
Chromosome

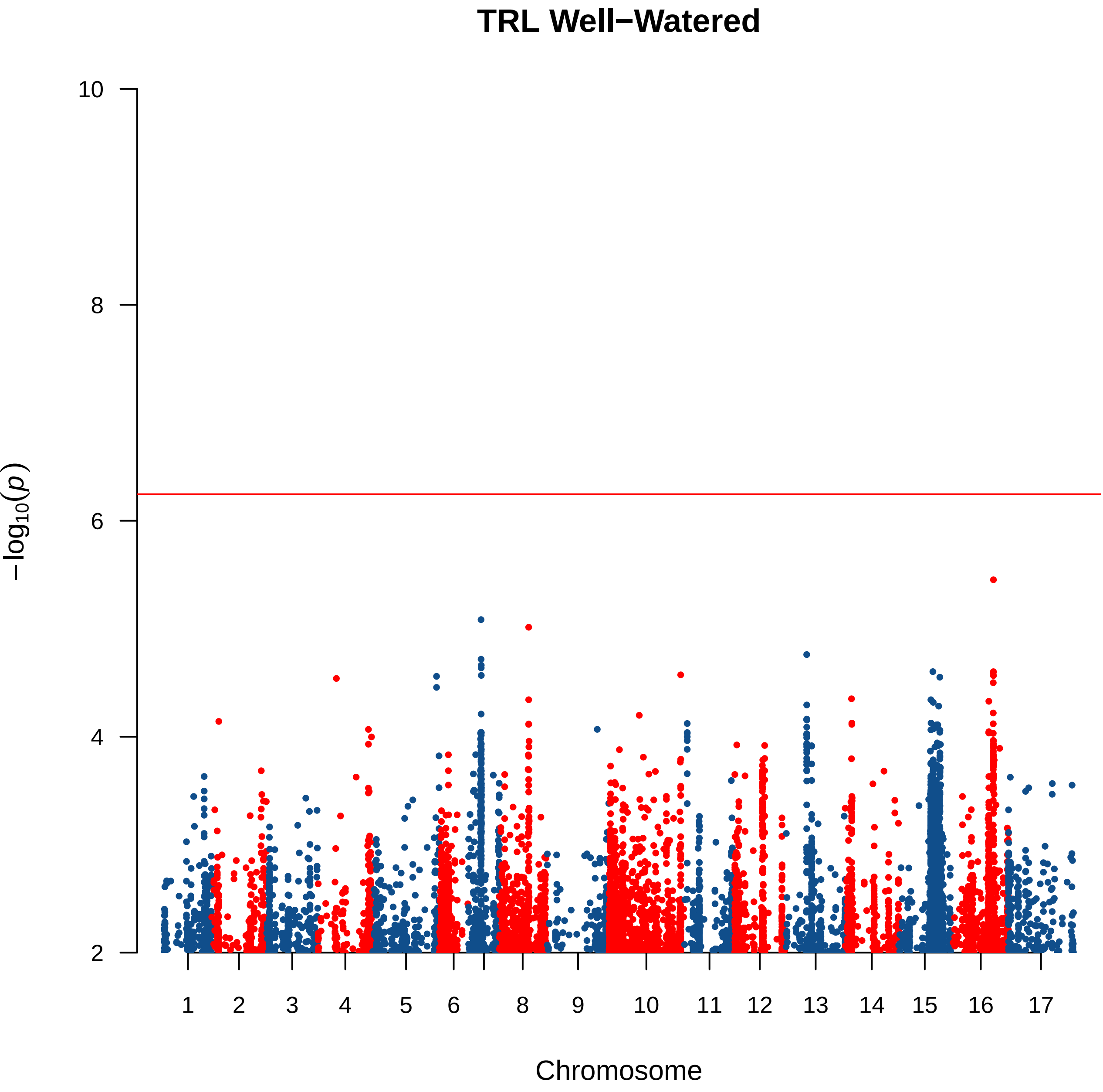
TRL Well−Watered
10
8
6
4
2
−log10(p)
1
2
3
4
5
6
8
9
10
11
12
13
14
15
16
17
Chromosome

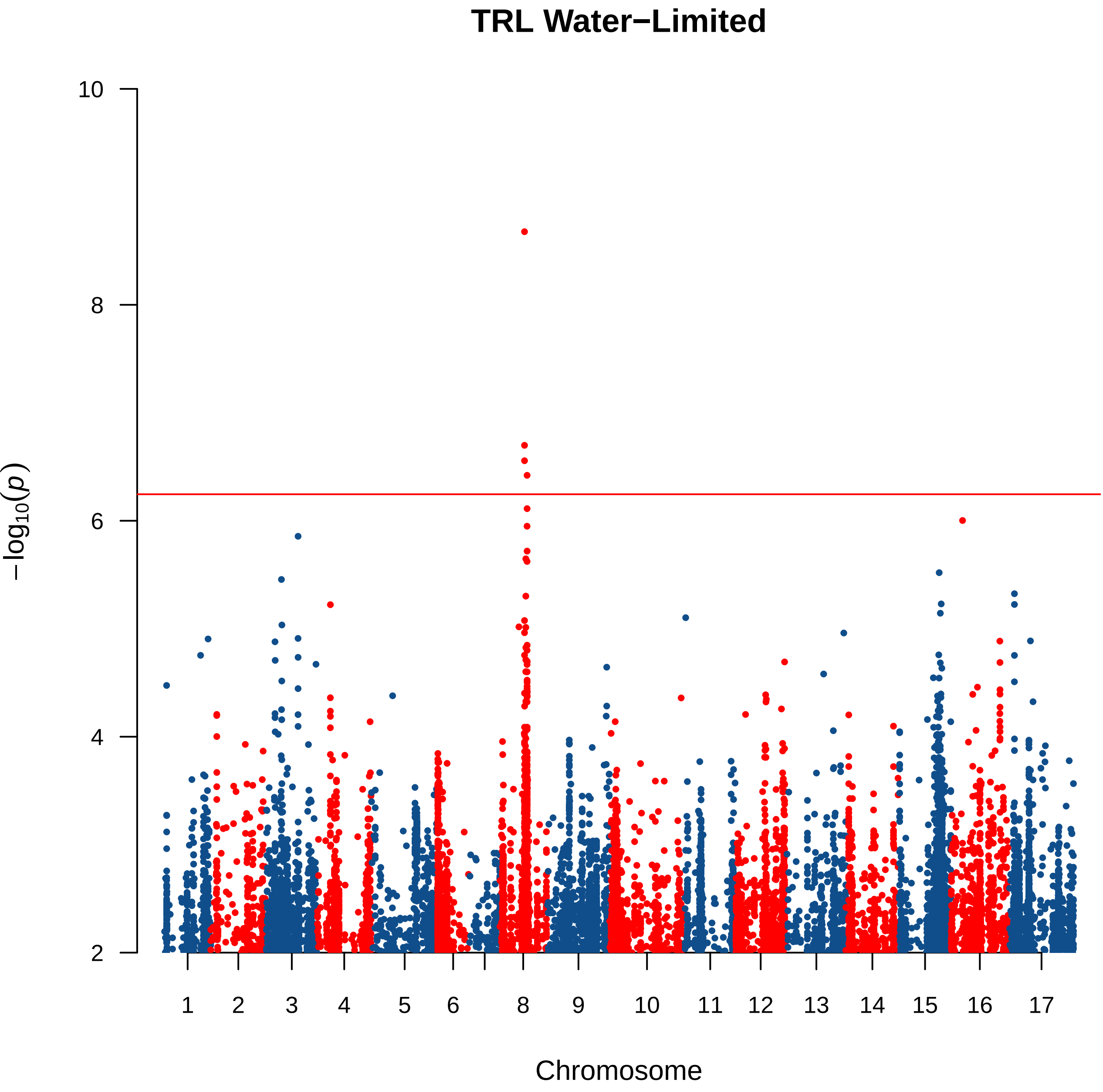
TRL Water−Limited
−log10(p)
10
8
6
4
2
1
2
3
4
5
6
8
9
10
11
12
13
14
15
16
17
Chromosome
