# Supplementary material for: Multiple genomic regions influence root morphology and seedling growth in cultivated sunflower (Helianthus annuus L.) under well-watered and water-limited conditions
Source: PLoS One. 2018 Sep 20;13(9):e0204279. doi: 10.1371/journal.pone.0204279 (PMC6147562; doi:10.1371/journal.pone.0204279)

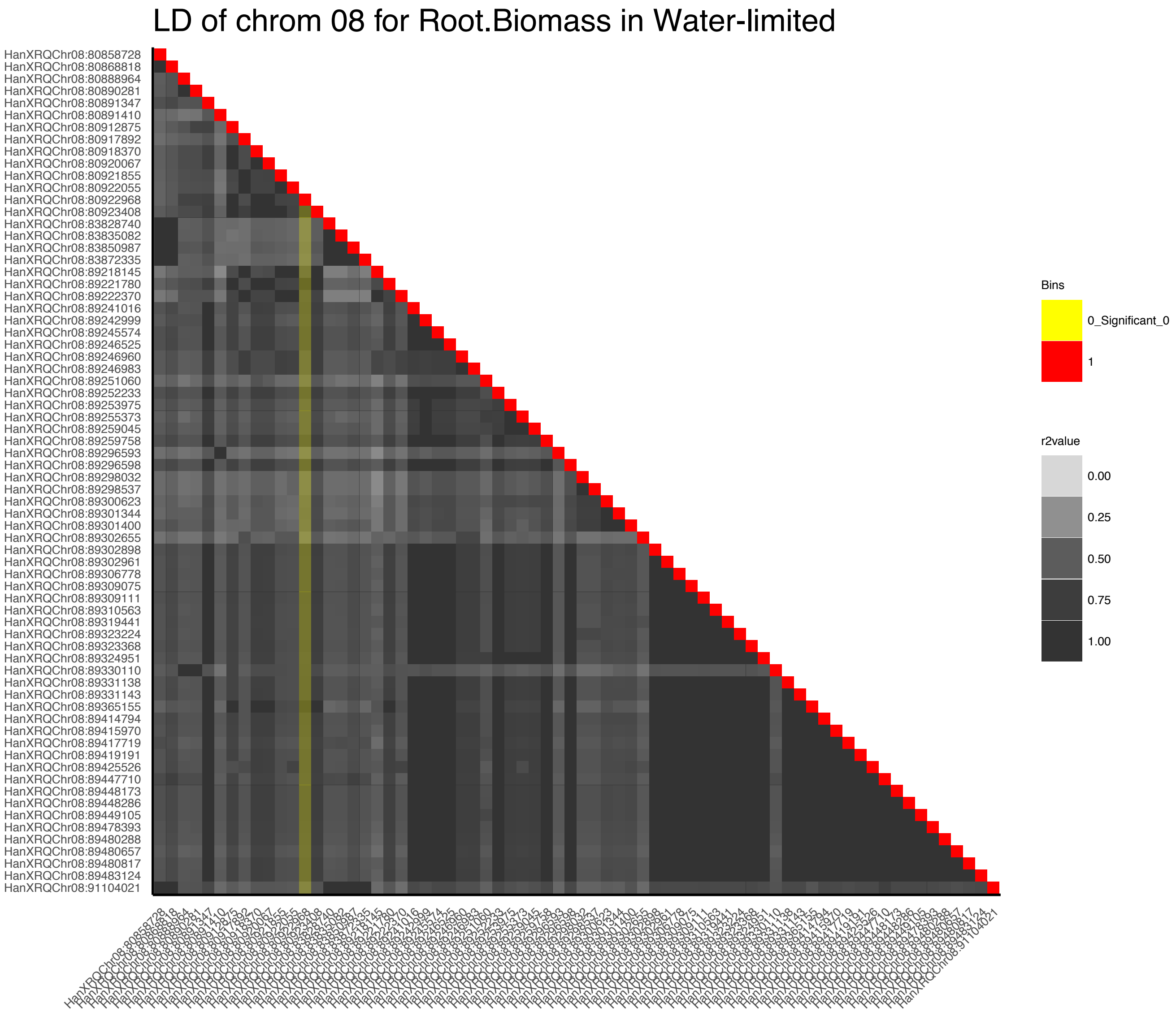
LD of chrom 08 for Root.Biomass in Water-limited
HanXRQChr08:80858728
HanXRQChr08:80868818
HanXRQChr08:80888964
HanXRQChr08:80890281
HanXRQChr08:80891347
HanXRQChr08:80891410
HanXRQChr08:80912875
HanXRQChr08:80917892
HanXRQChr08:80918370
HanXRQChr08:80920067
HanXRQChr08:80921855
HanXRQChr08:80922055
HanXRQChr08:80922968
HanXRQChr08:80923408
HanXRQChr08:83828740
HanXRQChr08:83835082
HanXRQChr08:83850987
HanXRQChr08:83872335
HanXRQChr08:89218145
HanXRQChr08:89221780
HanXRQChr08:89222370
HanXRQChr08:89241016
HanXRQChr08:89242999
HanXRQChr08:89245574
HanXRQChr08:89246525
HanXRQChr08:89246960
HanXRQChr08:89246983
HanXRQChr08:89251060
HanXRQChr08:89252233
HanXRQChr08:89253975
HanXRQChr08:89255373
HanXRQChr08:89259045
HanXRQChr08:89259758
HanXRQChr08:89296593
HanXRQChr08:89296598
HanXRQChr08:89298032
HanXRQChr08:89298537
HanXRQChr08:89300623
HanXRQChr08:89301344
HanXRQChr08:89301400
HanXRQChr08:89302655
HanXRQChr08:89302898
HanXRQChr08:89302961
HanXRQChr08:89306778
HanXRQChr08:89309075
HanXRQChr08:89309111
HanXRQChr08:89310563
HanXRQChr08:89319441
HanXRQChr08:89323224
HanXRQChr08:89323368
HanXRQChr08:89324951
HanXRQChr08:89330110
HanXRQChr08:89331138
HanXRQChr08:89331143
HanXRQChr08:89365155
HanXRQChr08:89414794
HanXRQChr08:89415970
HanXRQChr08:89417719
HanXRQChr08:89419191
HanXRQChr08:89425526
HanXRQChr08:89447710
HanXRQChr08:89448173
HanXRQChr08:89448286
HanXRQChr08:89449105
HanXRQChr08:89478393
HanXRQChr08:89480288
HanXRQChr08:89480657
HanXRQChr08:89480817
HanXRQChr08:89483124
HanXRQChr08:91104021
Bins
0_Significant_0
1
r2value
0.00
0.25
0.50
0.75
1.00

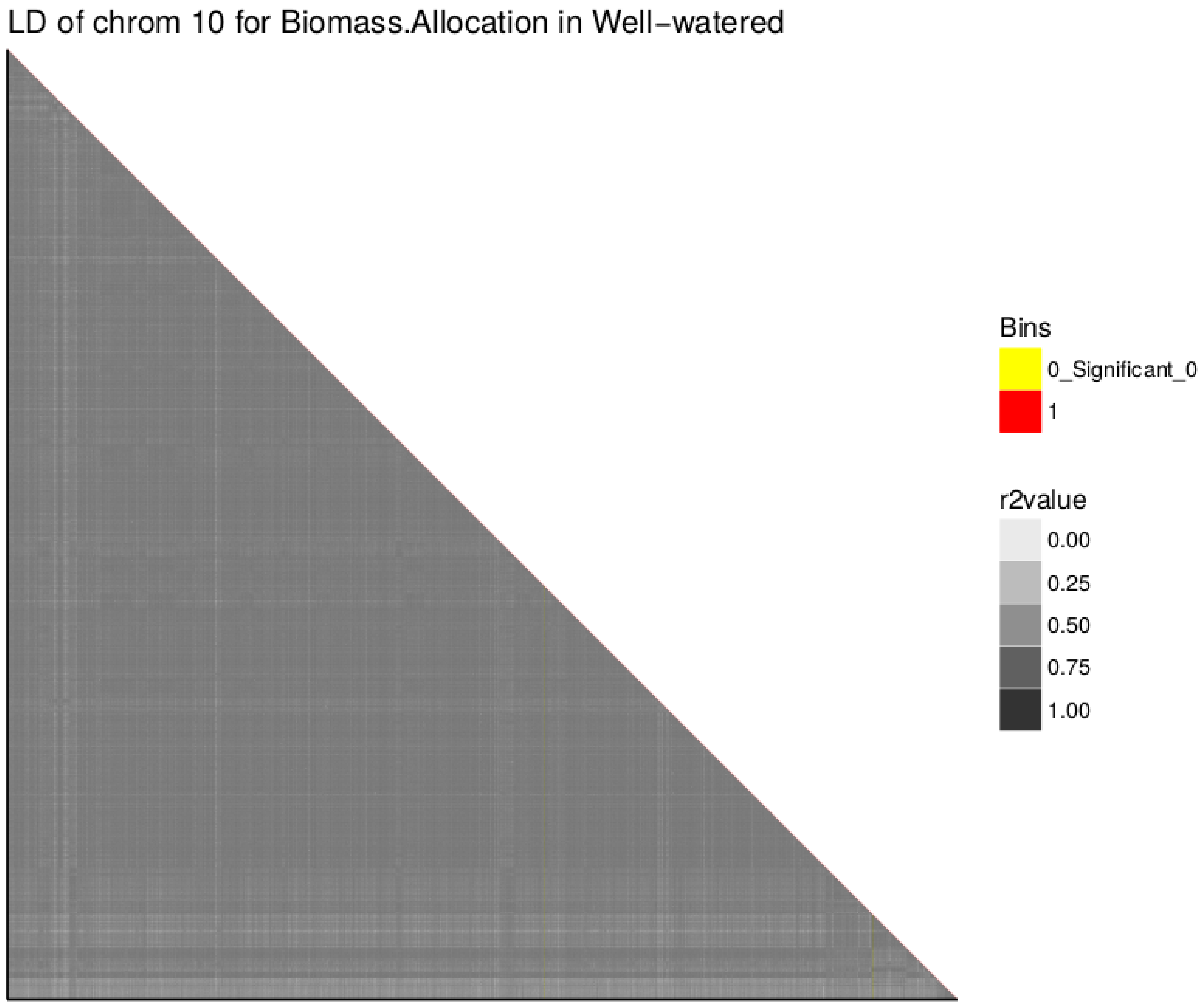
LD of chrom 10 for Biomass.Allocation in Well−watered
Bins
0_Significant_0
1
r2value
0.00
0.25
0.50
0.75
1.00

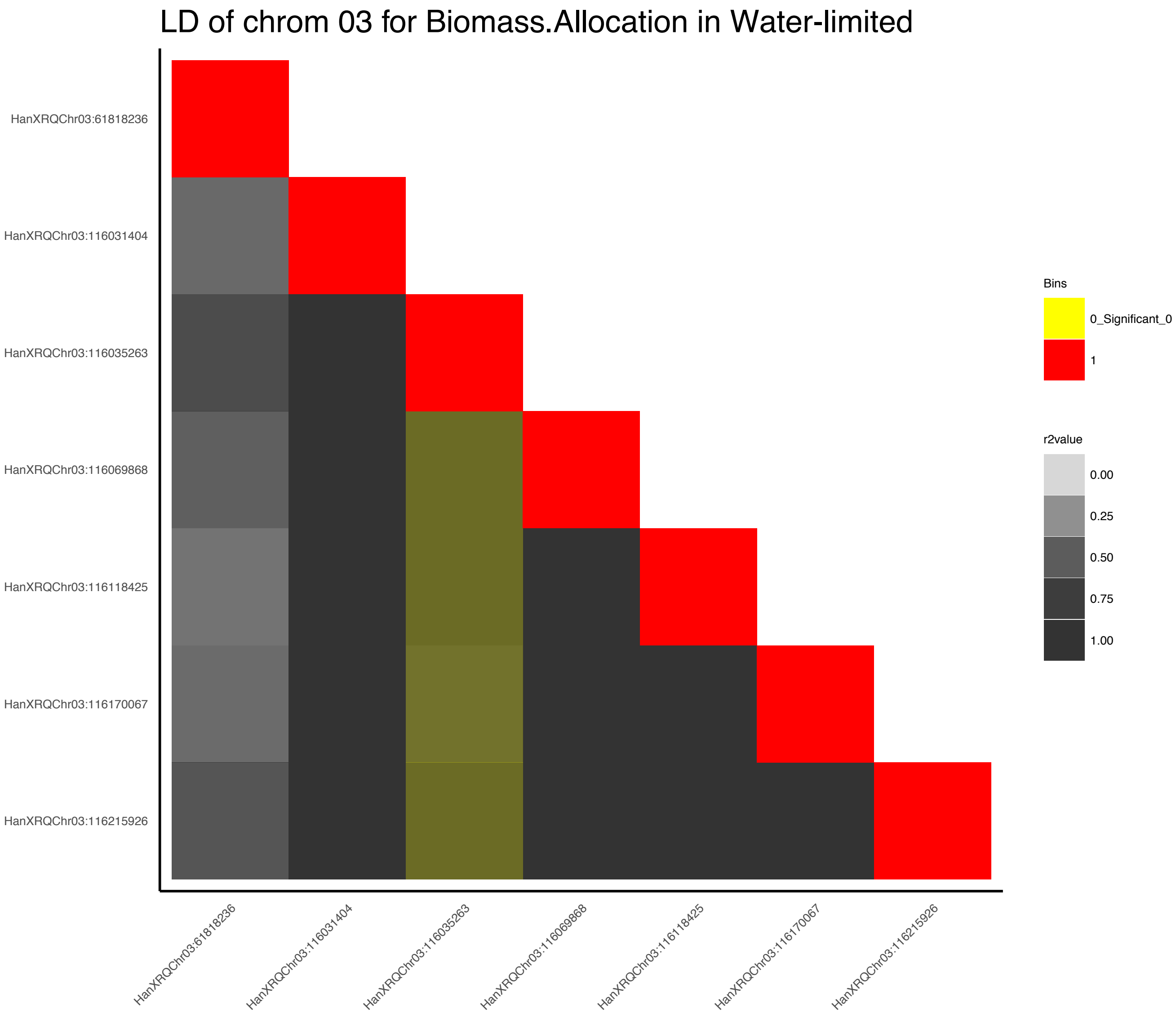

LD of chrom 03 for Biomass.Allocation in Water-limited
HanXRQChr03:61818236
HanXRQChr03:116031404
HanXRQChr03:116035263
HanXRQChr03:116069868
HanXRQChr03:116118425
HanXRQChr03:116170067
HanXRQChr03:116215926
Bins
0_Significant_0
1
r2value
0.00
0.25
0.50
0.75
1.00

LD of chrom 10 for Stem.Diameter in Water-limited

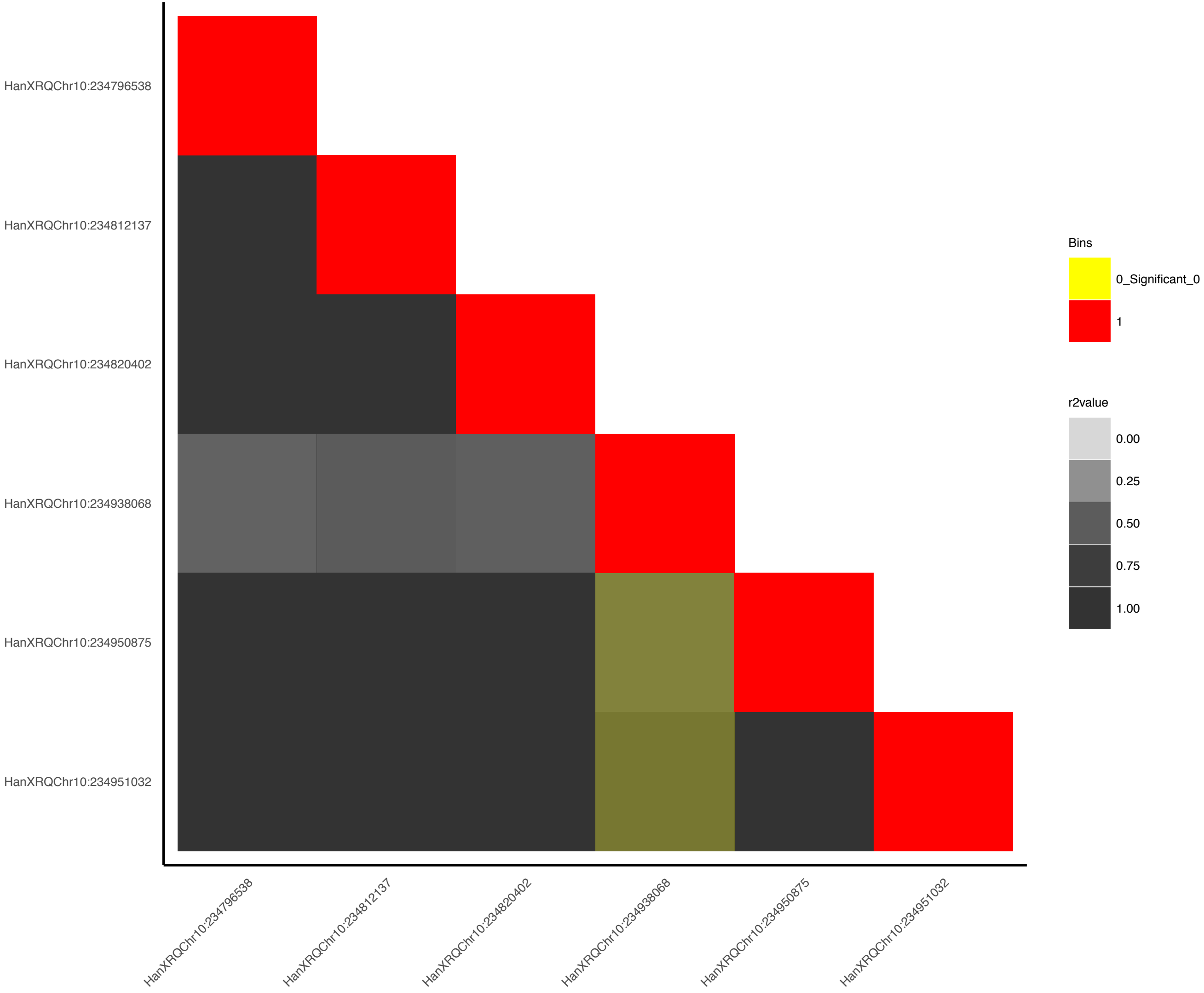

LD of chrom 12 for Stem.Diameter in Well-watered

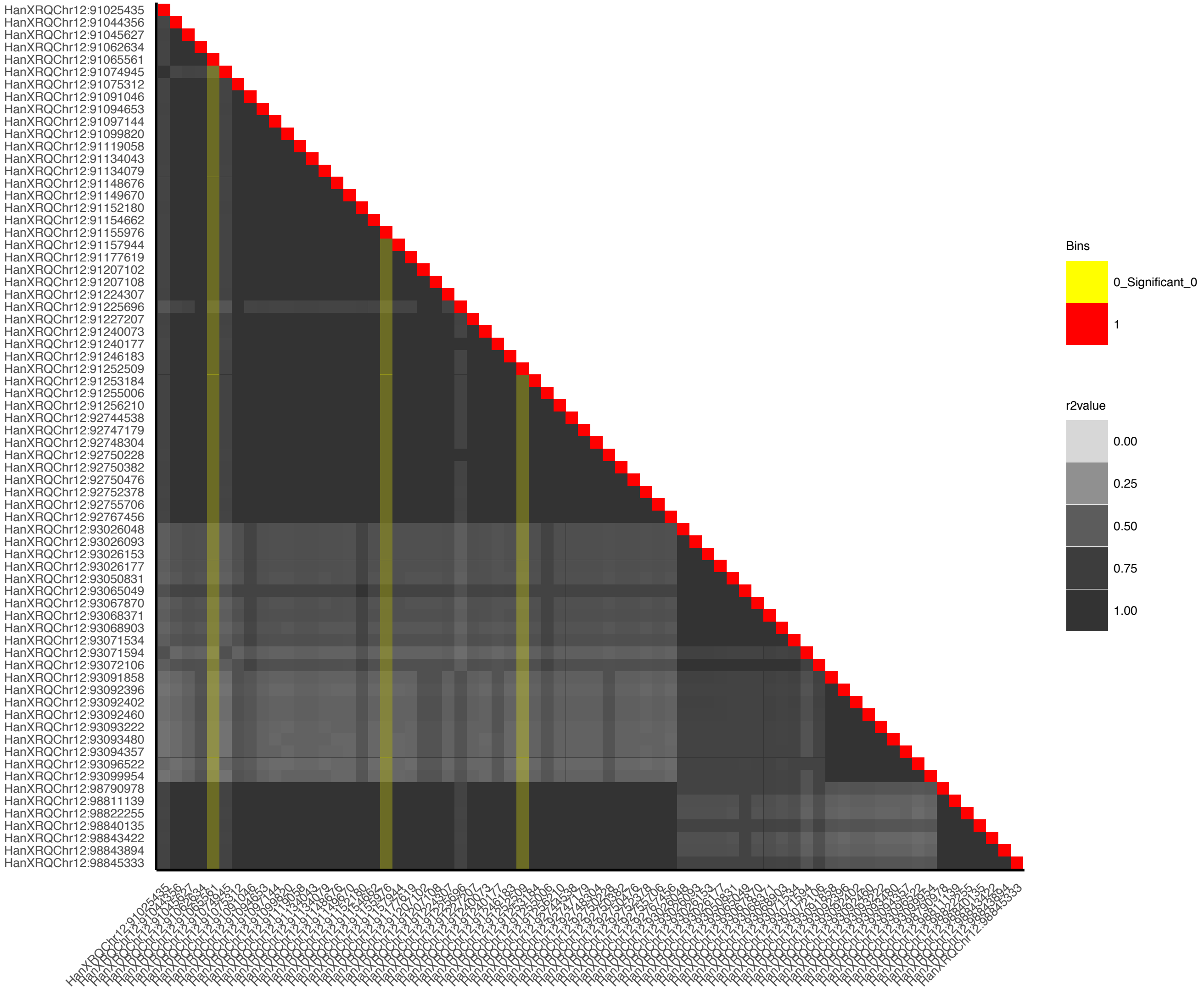

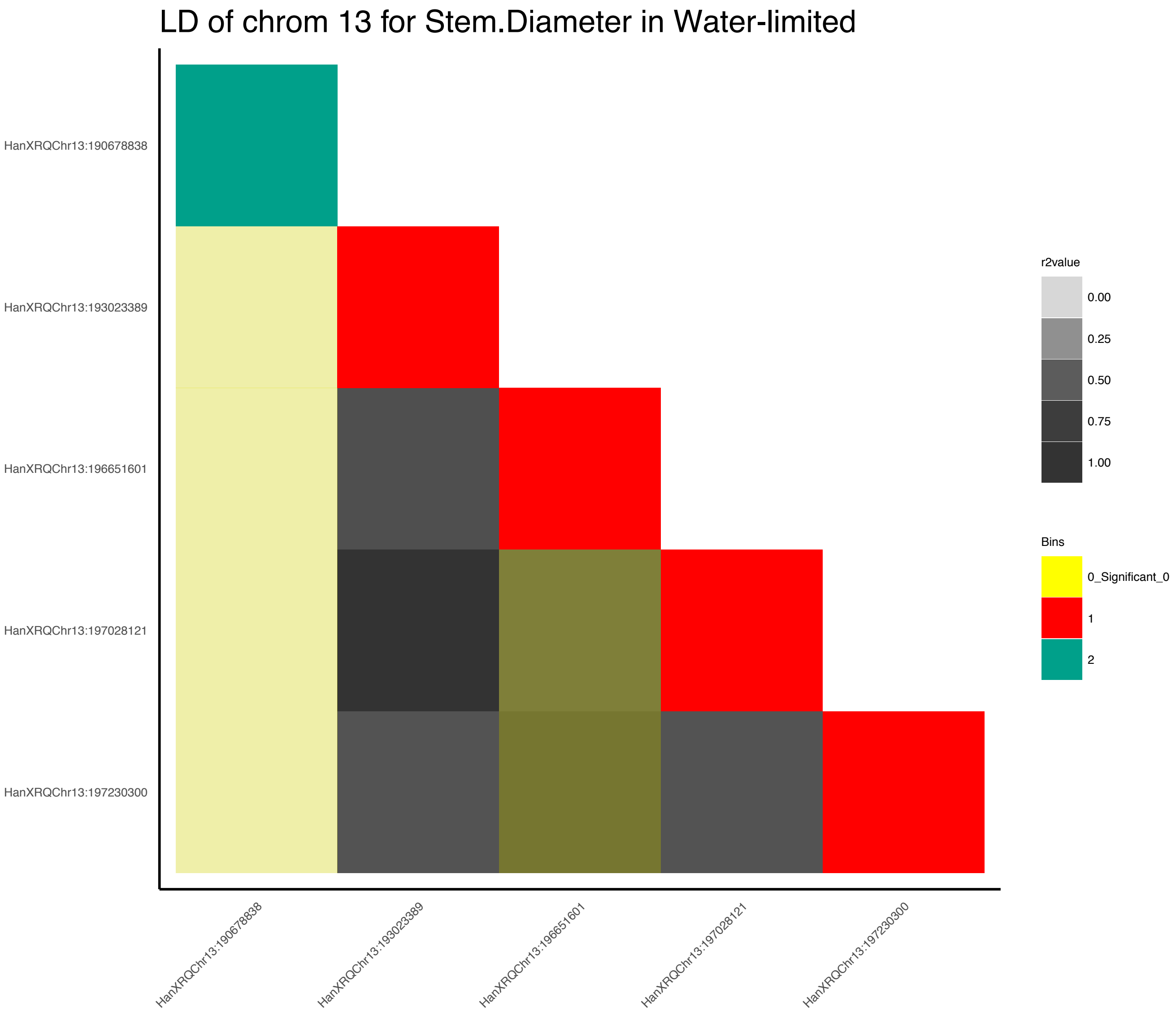
LD of chrom 13 for Stem.Diameter in Water-limited
HanXRQChr13:190678838
HanXRQChr13:193023389
HanXRQChr13:196651601
HanXRQChr13:197028121
HanXRQChr13:197230300
r2value
0.00
0.25
0.50
0.75
1.00
Bins
0_Significant_0
1
2

LD of chrom 14 for Stem.Diameter in Water-limited

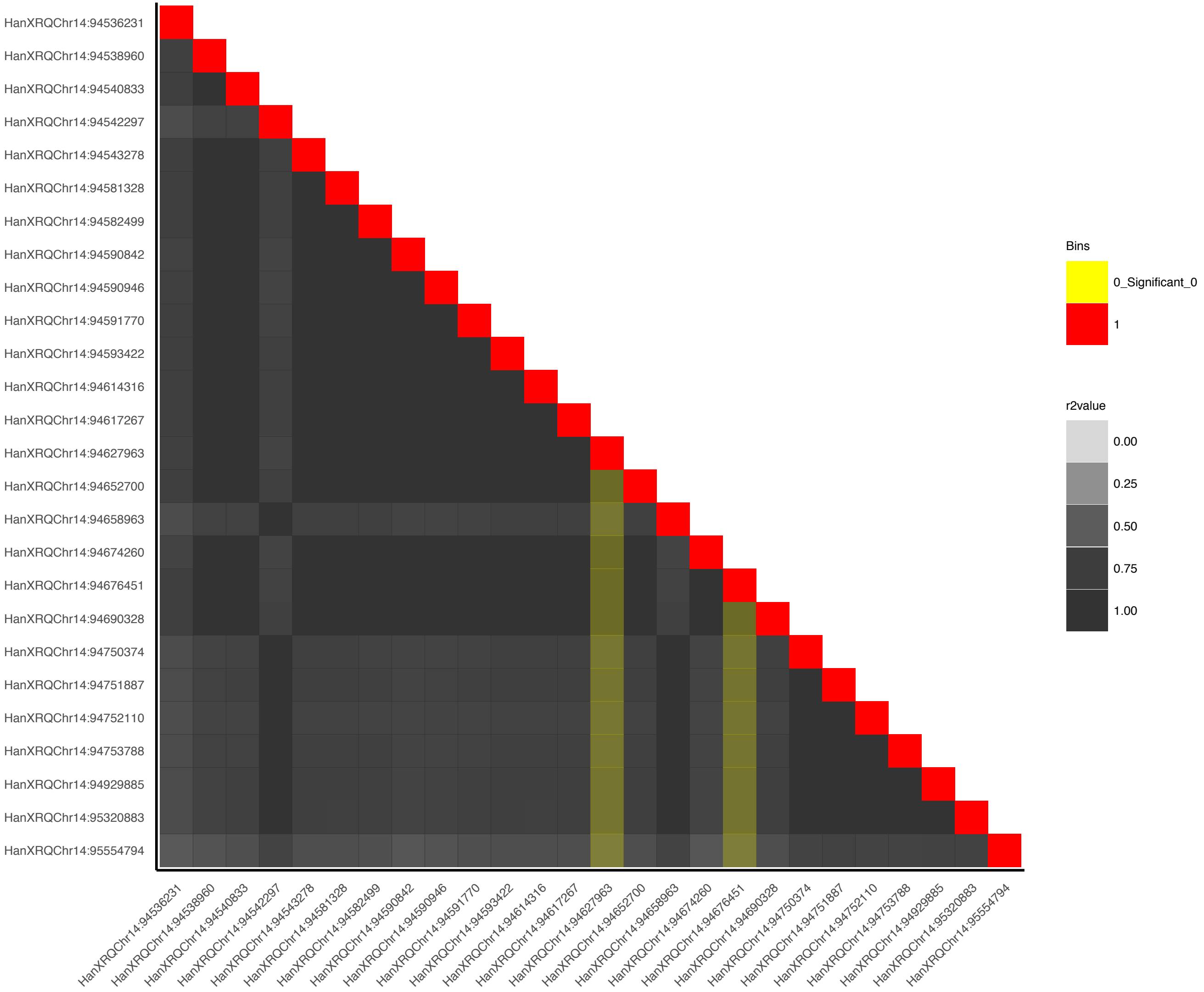

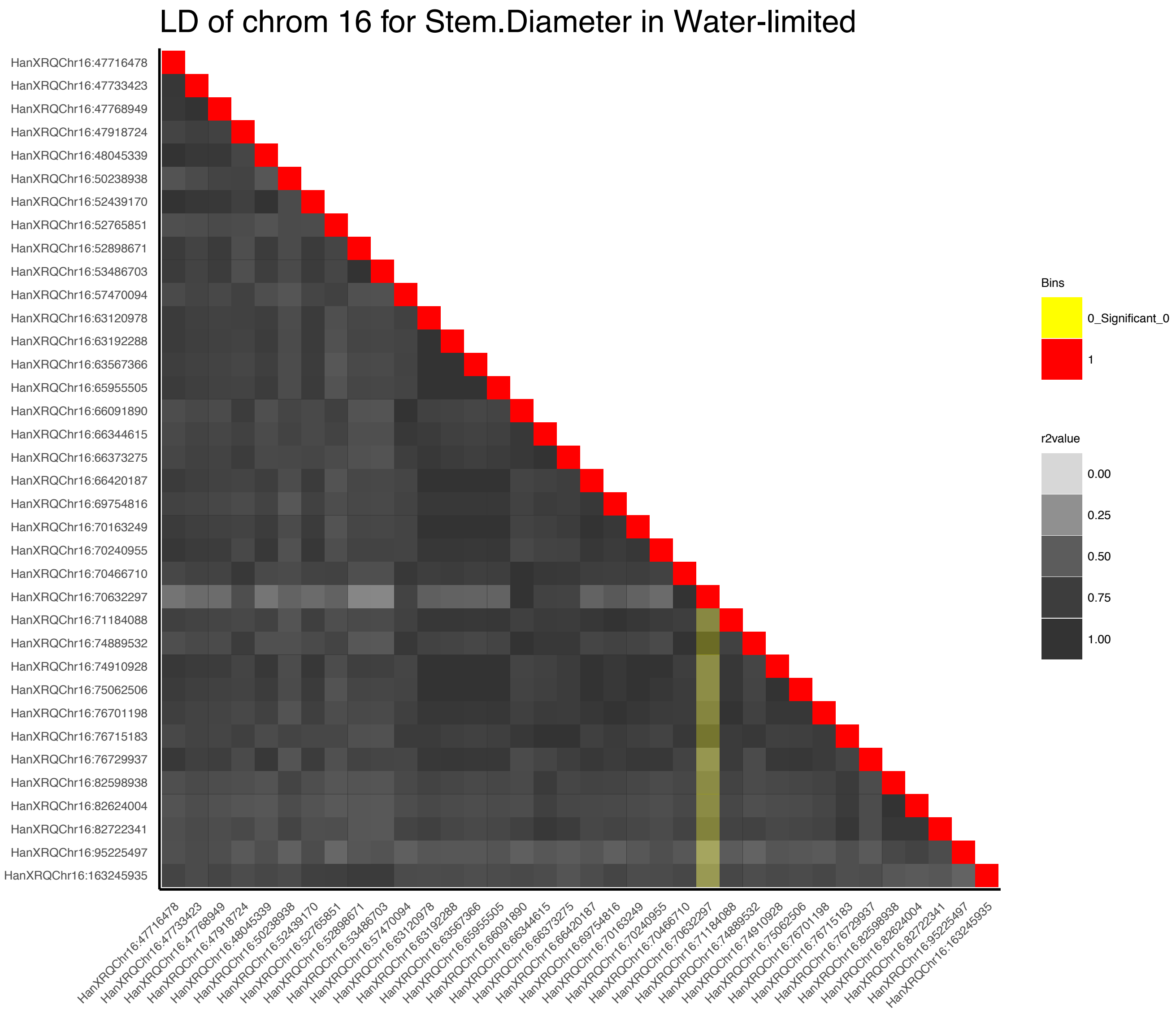
LD of chrom 16 for Stem.Diameter in Water-limited
HanXRQChr16:47716478
HanXRQChr16:47733423
HanXRQChr16:47768949
HanXRQChr16:47918724
HanXRQChr16:48045339
HanXRQChr16:50238938
HanXRQChr16:52439170
HanXRQChr16:52765851
HanXRQChr16:52898671
HanXRQChr16:53486703
HanXRQChr16:57470094
HanXRQChr16:63120978
HanXRQChr16:63192288
HanXRQChr16:63567366
HanXRQChr16:65955505
HanXRQChr16:66091890
HanXRQChr16:66344615
HanXRQChr16:66373275
HanXRQChr16:66420187
HanXRQChr16:69754816
HanXRQChr16:70163249
HanXRQChr16:70240955
HanXRQChr16:70466710
HanXRQChr16:70632297
HanXRQChr16:71184088
HanXRQChr16:74889532
HanXRQChr16:74910928
HanXRQChr16:75062506
HanXRQChr16:76701198
HanXRQChr16:76715183
HanXRQChr16:76729937
HanXRQChr16:82598938
HanXRQChr16:82624004
HanXRQChr16:82722341
HanXRQChr16:95225497
HanXRQChr16:163245935
Bins
0_Significant_0
1
r2value
0.00
0.25
0.50
0.75
1.00

LD of chrom 05 for Taproot.Length in Water-limited

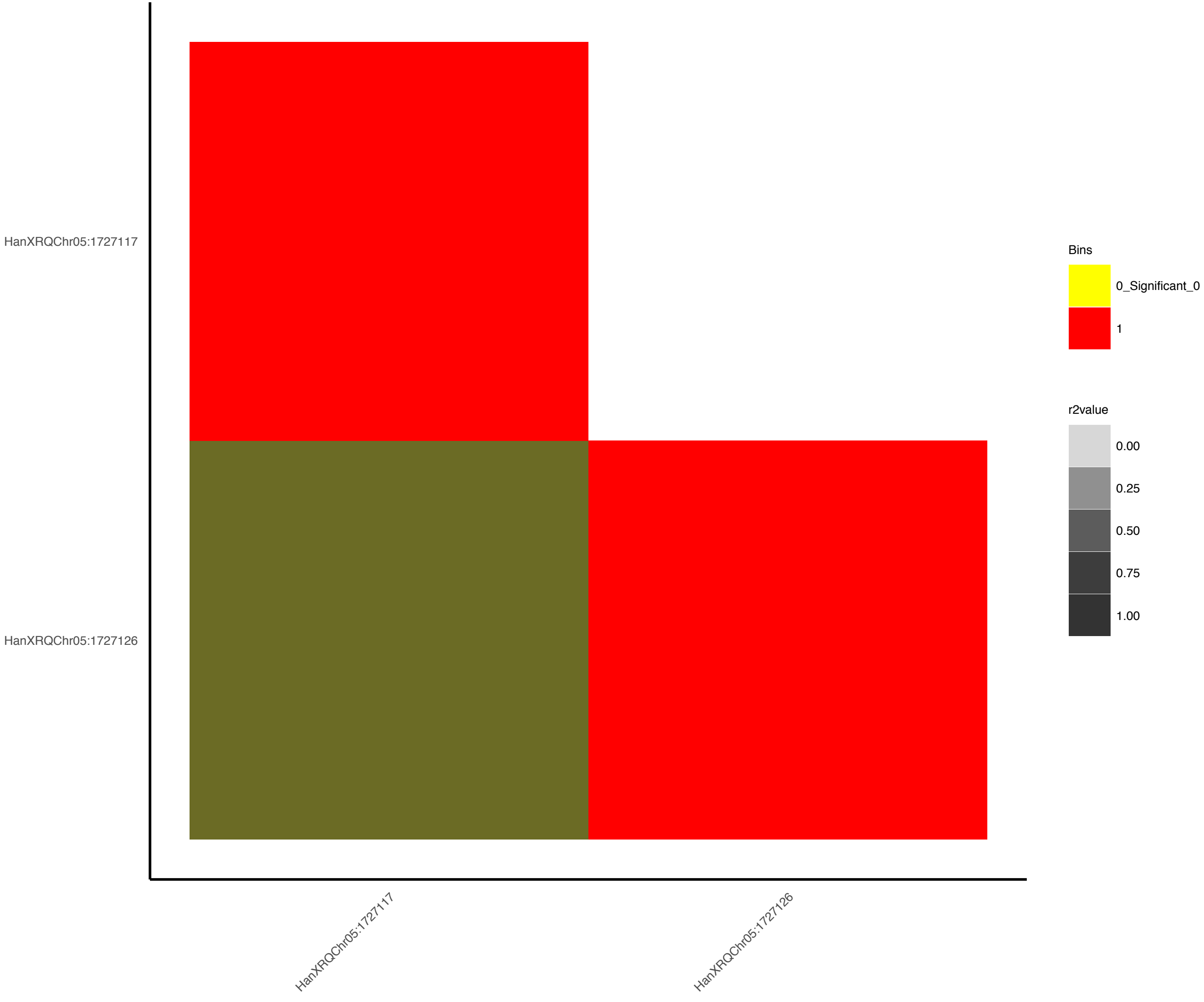

LD of chrom 08 for TRL in Water-limited

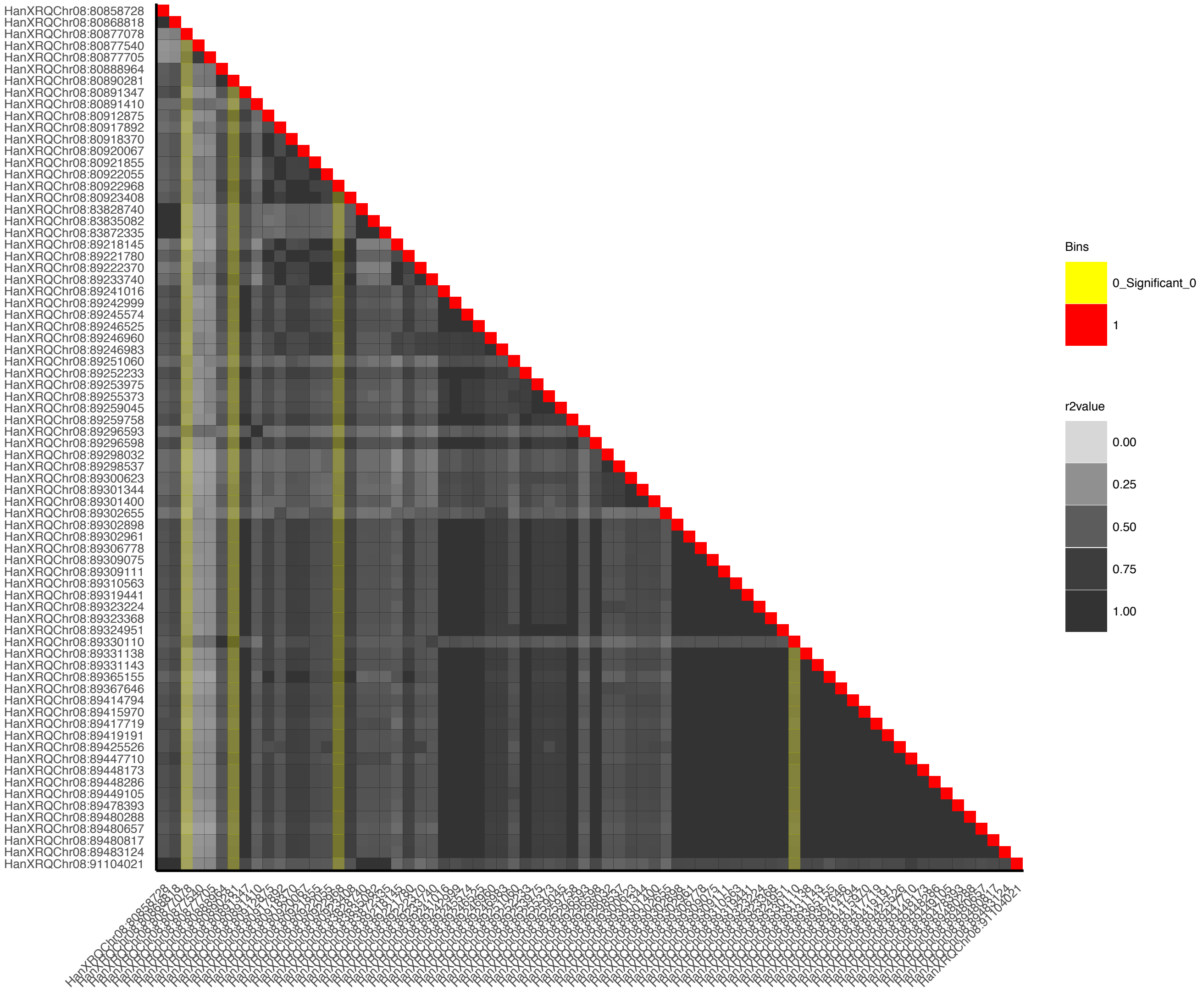

Supplement: S4 Fig — Colors along the diagonal indicate similar groups of markers based on r2. Plots separated per chromosome, per trait, per treatment. As described in the methods we used LDSelect to determine the boundaries of the region showing strong LD with significant SNPs. Significant SNPs are marked in yellow, all others are suggestive. (PDF) [file pone.0204279.s004.pdf]
